# Differential overexpression of *SERPINA3* in human prion diseases

**DOI:** 10.1038/s41598-017-15778-8

**Published:** 2017-11-15

**Authors:** S. Vanni, F. Moda, M. Zattoni, E. Bistaffa, E. De Cecco, M. Rossi, G. Giaccone, F. Tagliavini, S. Haïk, J. P. Deslys, G. Zanusso, J. W. Ironside, I. Ferrer, G. G. Kovacs, G. Legname

**Affiliations:** 1 Laboratory of Prion Biology, Department of Neuroscience, SISSA, Trieste, Italy; 2 Neurology and Neuropathology Unit, IRCCS Foundation Carlo Besta Neurological Institute, Milan, Italy; 3 Sorbonne Universités, UPMC Univ Paris 06 UMR S 1127, and CNRS UMR 7225, and ICM, Paris, France; 4 Atomic Energy Commission (CEA), DRF, Jacob, SEPIA, Fontenay-aux-Roses, France; 5 Department of Neurosciences, Biomedicine and Movement Sciences, University of Verona, Verona, Italy; 6 National CJD Research & Surveillance Unit, Centre for Clinical Brain Sciences, University of Edinburgh, Edinburgh, UK; 7 Institute of Neuropathology, Bellvitge University Hospital, IDIBELL; Department of Pathology and Experimental Therapeutics, University of Barcelona; CIBERNED; Hospitalet de Llobregat, Llobregat, Spain; 8 Institute of Neurology, Medical University of Vienna, Vienna, Austria

## Abstract

Prion diseases are fatal neurodegenerative disorders with sporadic, genetic or acquired etiologies. The molecular alterations leading to the onset and the spreading of these diseases are still unknown. In a previous work we identified a five-gene signature able to distinguish intracranially BSE-infected macaques from healthy ones, with *SERPINA3* showing the most prominent dysregulation. We analyzed 128 suitable frontal cortex samples, from prion-affected patients (variant Creutzfeldt-Jakob disease (vCJD) n = 20, iatrogenic CJD (iCJD) n = 11, sporadic CJD (sCJD) n = 23, familial CJD (gCJD) n = 17, fatal familial insomnia (FFI) n = 9, Gerstmann–Sträussler–Scheinker syndrome (GSS)) n = 4), patients with Alzheimer disease (AD, n = 14) and age-matched controls (n = 30). Real Time-quantitative PCR was performed for *SERPINA3* transcript, and *ACTB*, *RPL19*, *GAPDH* and *B2M* were used as reference genes. We report *SERPINA3* to be strongly up-regulated in the brain of all human prion diseases, with only a mild up-regulation in AD. We show that this striking up-regulation, both at the mRNA and at the protein level, is present in all types of human prion diseases analyzed, although to a different extent for each specific disorder. Our data suggest that *SERPINA3* may be involved in the pathogenesis and the progression of prion diseases, representing a valid tool for distinguishing different forms of these disorders in humans.

## Introduction

Prion diseases are rare, fatal and rapidly progressive neurodegenerative disorders. In humans these diseases may be sporadic, genetic or acquired^1^. Acquired forms in humans include variant Creutzfeldt-Jakob disease (vCJD) – caused by exposure to bovine spongiform encephalopathy (BSE) infected material – whose incidence has been progressively declining due to preventive measures^2^. However, the recent report of the first vCJD patient heterozygous at codon 129 in the prion protein gene (*PRNP*) creates some concerns about a second wave of the disease in the next few years^3^. In addition, three major studies about prion accumulation in lymphoid tissue conducted in a large series of appendix specimens carried out to date all show the prevalence of abnormal prion protein in the UK population to be around 1 in 2,000–1 in 5,000 using immunohistochemical (IHC) testing, suggesting a high prevalence of asymptomatic carriers^4–6^. Although safety procedures were put in place in the past decades to prevent the iatrogenic transmission of CJD, new cases of iCJD continue to be identified with incubation periods of several decades^7^. Moreover, probable iatrogenic transmission of vCJD through blood transfusion has been reported for four cases, from 2003 until 2009, posing additional public health concerns^8–11^. However, sporadic CJD (sCJD) still accounts for the majority of prion disease cases, which overall affect 1–2 persons per million of population worldwide^7^. The pathological agent responsible for these diseases is an aberrant misfolded isoform of the cellular prion protein (PrP^C^) denoted as prion or PrP^Sc^ ^12^. The latter represents the only specific biomarker for the disease, which is readily detectable in post-mortem central nervous system (CNS) tissues by Western blotting after limited proteolytic treatment^13^. Other neuropathological alterations are a prominent reactive astrocytic gliosis, involving activation of astrocytes, assessed by detection of increased levels of glial fibrillary acidic protein (GFAP), vacuolation (also known as spongiform change) and neuronal loss in grey matter. Altogether, the type of abnormal deposition of PrP^Sc^, astrocytic gliosis, and vacuolation in specific gray matter regions of the CNS are defining traits of different prion disorders^13^.

The prion protein is encoded by the *PRNP* gene located on chromosome 20. The gene contains 2 exons, with the entire coding region being contained in the last exon, thus excluding possible alternative splicing^12^. Human PrP^C^ is a protein of 253 amino acids, prior to post-translational modifications, and in its mature form is a 208 amino acid polypeptide, which is glycosylphosphatidylinositol-anchored to the outer leaflet of the cellular membrane with a unique primary sequence^14^. Misfolding of PrP^C^ into the pathogenic PrP^Sc^ occurs in different prion diseases, in which different forms of the protease-resistant core of PrP^Sc^ (sometimes referred as PrP^res^) can be identified. Different types of human prion diseases are characterized by diverse PrP^Sc^ isoforms and by the polymorphism present at *PRNP* codon 129. The two alleles of the gene can code for either methionine or valine in this position and homozygosity or heterozygosity can influence the pathological phenotypes in prion disorders. In addition, over 50 mutations in the *PRNP* open reading frame have been identified in genetic forms of prion diseases, in which the clinical and pathological features may also be influenced by the codon 129 polymorphism on the mutated and non-mutated alleles^1^.

In a recent analysis of the transcriptomic landscape in a model of prion infection using non-human primates, we identified a set of genes that are specifically dysregulated in the frontal cortex in the late stages of the disease^15^. In the present work we sought to validate whether up-regulated gene transcripts could be also dysregulated in human specimens of prion disease. We focused on *SERPINA3*, a gene encoding for a member of the serine protease inhibitor family of acute phase proteins^16^. Although serpins are predominantly produced in the liver, they are also synthesized in the brain, mainly by astrocytes and they have been reported to be involved in neuroinflammation and neurodegeneration^16^. Another member of the serpin superfamily named neuroserpin is predominantly expressed in neurons and its main function is to inhibit the enzyme tissue-type plasminogen activator. Point mutations in the neuroserpin protein determine a change in its conformation that leads to its abnormal cytoplasmic accumulation in neuroserpinopathies^17^. Concerning *SERPINA3*, it is a 56–66 kDa protein susceptible of heavy glycosylation at multiple sites^18^. Interestingly, it is the only serpin able to bind DNA, probably through lysine repeat motifs in regions 210–212 and 391–396^19^. Its dysregulation has been already linked to a number of different diseases, such as chronic obstructive pulmonary disease^20^, cystic fibrosis^21^, pancreas^22^, prostate^23^, lung^24^ and invasive breast cancer^25^ and Alzheimer disease (AD)^26–29^.

BSE infection in humans results in the acquired prion disease vCJD which has also been modelled experimentally in primates. However, given the limited numbers of reported vCJD patients (231 worldwide, including three from Italy), we decided to extend our analysis to sCJD patients. This would also allow us to detect differences in gene regulation between acquired and sporadic human prion disorders. We also included in our analysis cases of iCJD caused by treatment with prion-contaminated human growth hormone (hGH). Moreover, we included patients with genetic forms of prion disease resulting from different *PRNP* mutations, in particular FFI (with the D178N mutation), GSS (two with the P102L mutation, one with the P84S mutation and one with the Q212P mutation) and gCJD (11 with the E200K mutation, one with the E211Q mutation, one with the V210I mutation and three cases with 4 or 5 octarepeat region insertions). Regarding control samples, we used age- sex- and size-matched control groups for each disease analyzed. In order to enable the identification of prion-specific gene expression alterations, we decided to introduce in our study also some samples from patients with non-CJD neurodegenerative disorder (AD) as an additional control group. We collected over 200 samples of frontal cortex of post-mortem specimens from various forms of prion diseases, AD and age-matched controls, of which only 128 resulted of suitable quality for transcriptomic analysis. The different samples included vCJD (n = 20), sCJD (n = 23), iatrogenic CJD (iCJD, n = 11), familial CJD (gCJD, n = 17), fatal familial insomnia (FFI, n = 9), Gerstmann–Sträussler–Scheinker (GSS) syndrome (n = 4), AD (n = 14) and age-matched controls (n = 30).

Real Time-quantitative PCR (RT-qPCR) was performed for the *SERPINA3* transcript, and four different housekeeping genes (*ACTB*, *RPL19*, *GAPDH* and *B2M*) were chosen as reference genes. We found strong up-regulation in the gene expression of *SERPINA3* in all prion diseases considered. Limited up-regulation for this transcript was also observed in AD samples, although not comparable to that of prion-affected samples. In addition, we investigated *GFAP* and *PRNP* genes expression levels in all patients considered. As for *GFAP*, we found a similar dysregulation pattern as for *SERPINA3* among all groups, up-regulation in all prion diseases together with a negligible increase in AD samples. Concerning *PRNP* transcriptional levels, after accurate analysis to rule out the influence of contaminating blood, a decrease in both iCJD and AD samples was observed. Nevertheless, these changes were limited when compared to *SERPINA3* transcript. Immunohistochemistry and biochemical analyses for SERPINA3 protein were also performed, confirming the specific and differential up-regulation of the protein in sporadic, genetic or acquired forms of prion disease.

## Results

### qPCR and WB analysis

**Figure 1:**
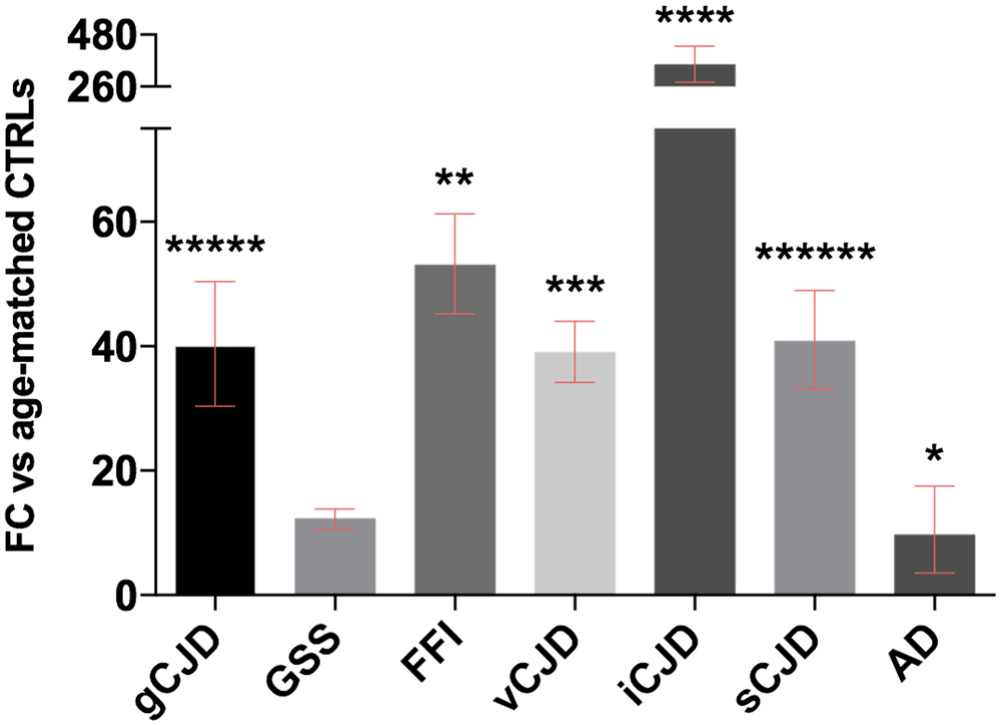
RT-qPCR analysis for *SERPINA3* mRNA expression in brain normalized against *GAPDH*. gCJD (n = 17) *****p < 0.000000005, FFI (n = 9) **p < 0.0001, vCJD (n = 20) ***p < 0.000001, iCJD (n = 11) ****p < 0.00000005, sCJD (n = 23) ******p < 0.0000000005, AD (n = 14) *p < 0.005). Fold Change (FC) values are presented as average with CI.

Two common issues with postmortem human brain samples are the stability of endogenous reference genes and the RNA integrity^30^. A number of different variables correlates to different extents with RNA integrity: for example, higher degradation is observed in samples subjected to prolonged thawing^31^ or storage^32^ as well as in samples of patients showing longer agony before death (probably due to brain acidosis)^33^. In studies involving postmortem human tissue is not possible to control all the variables that might impair RNA quality, and this inevitably leads to non-homogenous sample collections with high degree of biological variance. However, it is widely accepted that moderate degradation does not preclude reliable analyses of small amount of RNA. Indeed, it has been shown that gene expression profiles from partially degraded RNA samples with still visible ribosomal bands are highly similar to that of intact samples^32^, especially if the RT-qPCR amplified products are smaller than 250 bp^34^. This is not particularly surprising considering that reference genes transcripts most likely degrade gradually together with target ones^30^. For all these reasons, a RIN ≥ 4 was suggested as minimal threshold for human brain tissue^35^. In order to ensure a sufficient reliability of the samples, among the 225 samples collected, only 126 samples harbor RNA that showed RIN ≥ 4 or higher, and with the exception of few rare samples, were selected for further analysis, Regarding housekeeping genes, there is a lack of consensus about the optimal reference genes in postmortem brain tissues. Indeed, while *GAPDH* is the most widely used reference gene for transcriptomic studies, some issues have been reported about its involvement in human neurodegenerative processes^36^. However, *ACTB*, *B2M* and the ribosomal protein family seem to show good stability across different RIN values^33^ and brain samples^36^. Therefore, we decided to use *GAPDH*, *B2M*, *ACTB* and *RPL19* as reference genes to normalize RT-qPCR data, in order to increase the robustness of our findings. The relative mRNA levels, expressed as the fold change (FC), were calculated using the 2^−∆∆C^_T_ method and four reference genes, obtaining very similar results. Firstly, the four housekeeping genes were analyzed across diseased and control patients in order to assess their expression stability (Fig. 1S).

*SERPINA3* was found to be strongly up-regulated in all prion diseases: sCJD (FC = 40.3), vCJD (FC = 38.8), FFI (FC = 52.7), gCJD (FC = 39.1) and GSS (FC = 12.17, not significant) cases (Table 1). This up-regulation is particularly pronounced in iCJD patients (FC = 347) while, in comparison, only a modest up-regulation was observed in AD cases (FC = 6.7) (Fig. 1). Regarding GSS, we have to stress that the lack of significance in this group is likely due to the sample size (n = 4) which is too small to allow reliable statistical analysis. Regarding AD patients, we have to mention that the mean age of this group was higher (68.7 years) than that of the oldest control group available in this study (63.3 years). In addition, we observed that *SERPINA3* mRNA levels were increasing with age in non-neurodegenerative controls (CTRL group with mean 51yrs vs CTRL group with mean 29yrs FC = 3, CTRL group with mean 64yrs vs CTRL group with mean 29yrs FC = 7). Therefore, the limited up-regulation we detected in AD samples might be overestimated due to the lack of appropriate age-matched controls.

**Table 1:** Summary of sample groups used in the study.

Group	Number (n =)	*SERPINA3* FC	p value
CTRL	30	—	—
sCJD	23	40.28	0.0000000003
GSS	4	12.17	0.0870036245
FFI	9	52.66	0.0000648544
gCJD	17	39.12	0.0000000019
vCJD	20	38.80	0.0000001227
iCJD	11	347.07	0.0000000458
AD	14	6.72	0.0023142762

Values presented are normalized against *GAPDH*.

**Figure 2:**
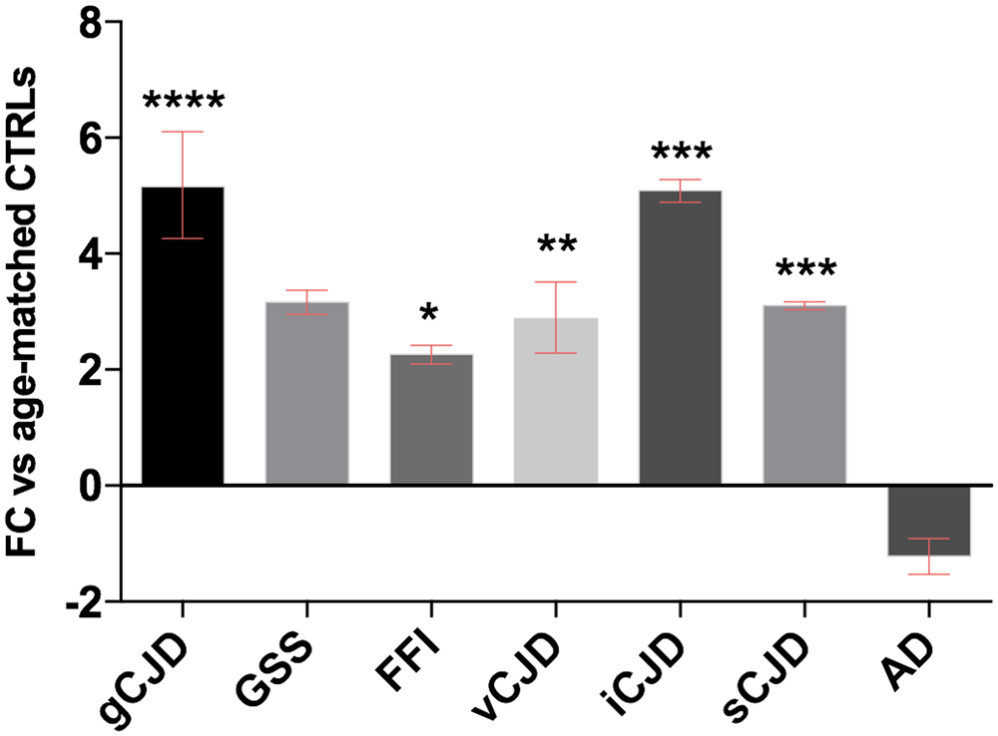
RT-qPCR analysis for GFAP mRNA expression in brain normalized against *GAPDH*. gCJD (n = 17) ****p ≤ 0.000001, FFI (n = 9) *p < 0.05, vCJD (n = 20) **p < 0.001, iCJD (n = 11) ***p < 0.0005, sCJD (n = 23) ***p < 0.0005. Fold Change (FC) values are presented as average with CI.

**Figure 3:**
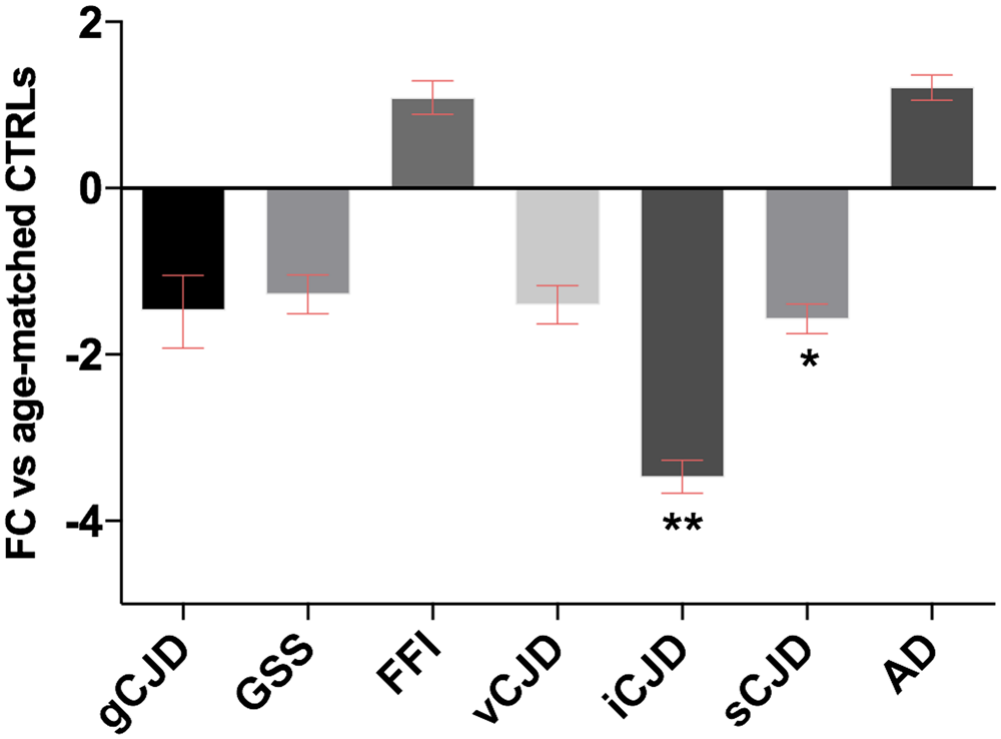
RT-qPCR analysis for *PRNP* mRNA expression in brain normalized against *GAPDH*. iCJD (n = 11) **p < 0.0005, sCJD (n = 23) *p < 0.05. Fold Change (FC) values are presented as average with CI.

**Figure 4:**
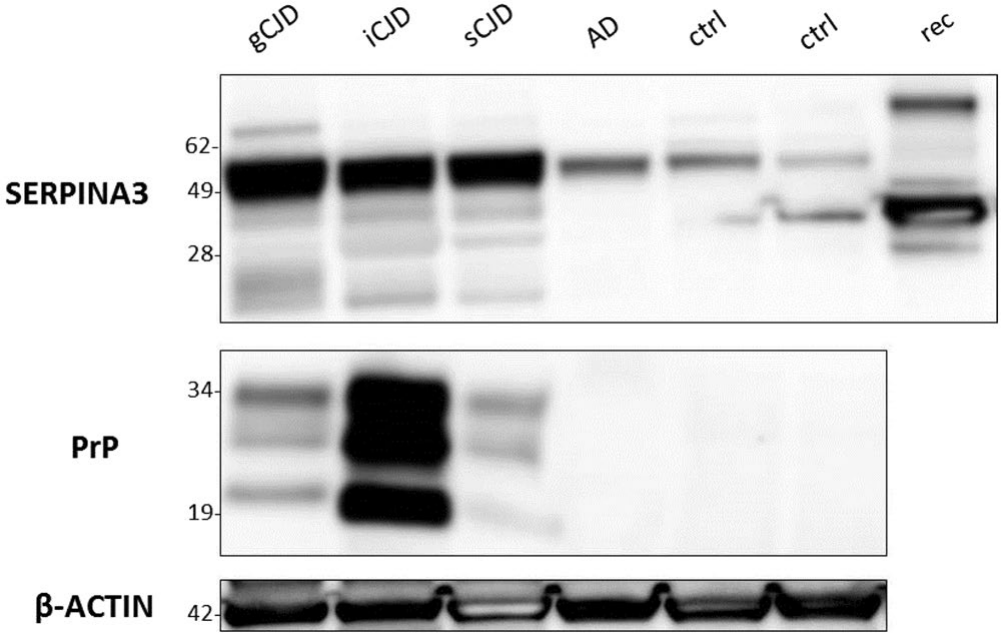
Western blotting analysis of brain samples from CJD, AD patients and healthy controls. The expression levels of SERPINA3 and PrP^Sc^ in brain samples of representative cases from each group were assessed by means of Western blotting, on two different gels run in parallel within the same experiment. β-actin was used as proteins loading control and to normalize the expression level of SERPINA3 for densitometric analysis. SERPINA3 and β-actin were developed on the same membrane, sequentially. SERPINA3 image has been cropped to improve clarity. Rec lane refers to recombinant SERPINA3 used as control. Molecular weight is represented on the left (kDa). Original full-length images are provided in Supplementary Material.

Very similar results were obtained against all four reference genes (Figs 2S, 3S and 4S). As regards healthy controls, *SERPINA3* mRNA was present at very low levels, in some cases barely detectable, with an average Ct of 31.2. The levels of *SERPINA3* mRNA in controls was then used as zero value to normalize all the others groups.

Dealing with prion disease in which astrogliosis is a prominent feature, we assessed also the mRNA levels of *GFAP* in all our samples. As for *SERPINA3*, *GFAP* was up-regulated in all prion diseases, with the highest value observed in gCJD patients (FC = 5.2) and iCJD (FC = 5), sCJD (FC = 3), GSS (FC = 2.9, not significant) vCJD (FC = 2.8) and FFI (FC = 2.2). No changes were observed in AD samples (Fig. 2).

In addition, we investigated the gene expression pattern of *PRNP*. Also for this gene, the greatest change was observed in the iCJD group, where *PRNP* mRNA is strongly down-regulated. Similarly, sCJD patients displayed a significant but moderate down-regulation (Fig. 3).

**Figure 5:**
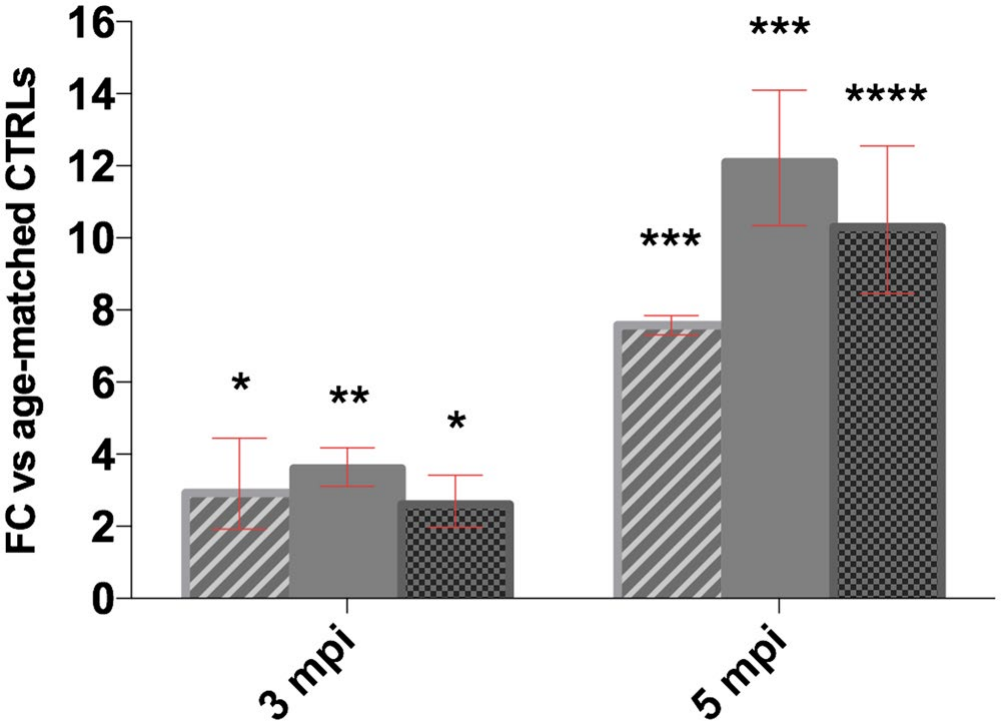
RT-qPCR analysis for *serpina3n* in the brain of RML-infected CD1 mice (n = 4 for each time point) *p < 0.05, **p < 0,005, ***p < 0.0005, ****p < 0.0001. Dashed column vs Gapdh, plain gray vs Tubb3, dotted column vs Actb. Fold changes are presented averaged with CI.

**Figure 6:**
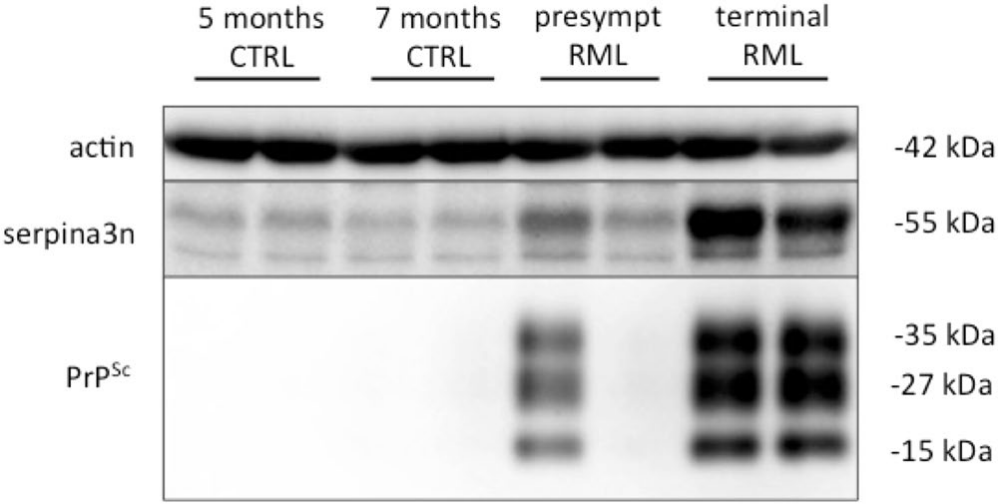
Western blotting analysis for serpina3n in the brain of RML-infected CD1 mice and related age-matched controls. The expression levels of serpina3n and PrP^Sc^ were assessed by means of Western blotting, on two different gels run in parallel within the same experiment. β-actin was used as proteins loading control and to normalize the expression level of serpina3n for densitometric analysis. serpina3n and β-actin were developed on the same membrane, sequentially. serpina3n image has been cropped to improve clarity. Molecular weights are shown on the left. Original full-length images are provided in Supplementary Material.

Finally, we analyzed all the samples for expression of an erythrocyte specific marker, *ALAS2*, to evaluate blood contamination in the various samples. RT-qPCR analysis of *ALAS2* revealed a small blood presence (C_T_ < 35) in the majority of samples (Fig. 5S). However, when investigating the possible presence of *SERPINA3*, *GFAP and PRNP* mRNA in the blood, only a very low signal for *PRNP* was detected, confirming that the blood contamination did not influence our analysis (Fig. 6S).

**Figure 7:**
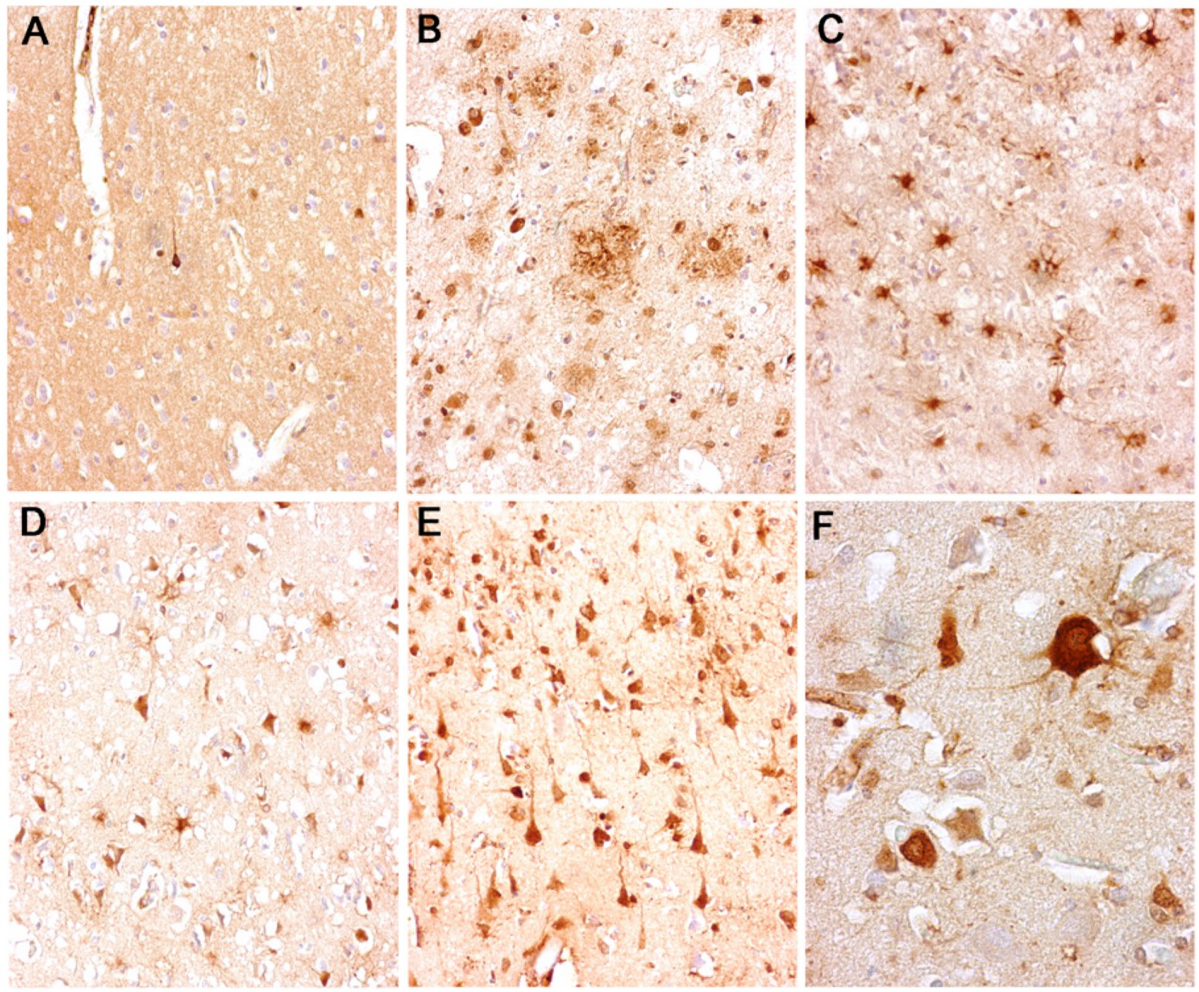
Neuropathological analysis. Immunohistochemical analysis revealed strong SERPINA3 immunoreaction within residual neurons and reactive glial cells in the frontal cortex of CJD patients [variant (**E,F**), sporadic (**D**) and iatrogenic (**C**)] while it was scanty and limited to isolated neurons in control subjects (**A**). In AD patients, anti-SERPINA3 decorated some cortical neurons and the amyloid of senile plaques (**B**).

After normalization against β-actin levels, Western blot analysis revealed the presence of higher level of SERPINA3 in all CJD brains (regardless the disease aetiology) if compared to that of AD and healthy controls. Interestingly, lower molecular weight bands – likely representing poorly glycosylated isoforms- are visible only in prion-infected samples. The presence of PrP^Sc^ was confirmed in all CJD brains (Fig. 4). According to densitometric analysis, the expression levels of SERPINA3 in CJD samples were six-fold higher than those of controls (Fig. 7S).

As for other tissues outside the brain, we also investigated the presence of SERPINA3 protein in the CSF where it is expressed, but with no significant differences found between AD and sCJD samples (Fig. 8S).

As for human brain samples, also RML-infected CD1 mice displayed a strong up-regulation of *serpina3n* at symptomatic stage, both at the mRNA and protein level (Figs 5, 6 and 9S). Even more importantly, this up-regulation was observed at the preclinical stage, suggesting the direct correlation between *serpina3n* and prion infection.

### Neuropathological analysis

Immunohistochemical analysis revealed that in the frontal cortex of control subjects, immunostaining for SERPINA3 was present in isolated neurons and not in other cell types (Fig. 7A). On the contrary, in CJD brain samples, either sporadic, iatrogenic or variant, most of the residual cortical neurons and numerous reactive astrocytes were intensely immunostained by anti-serpina3 antibody. In neuronal cells, the immunodecoration was most evident in the perikarya and in the apical dendrites of pyramidal neurons (Fig. 7C–F). PrP focal deposits (perivacuolar, perineuronal or plaque-like) were not immunoreactive. A consistent number of SERPINA3-immunoreactive cortical neurons was found in AD samples where the amyloid of senile plaques and amyloid angiopathy (not shown) was also immunopositive.

We also investigated the correlation between SERPINA3 and GFAP immunoreactivity. While a strong parallel between the two proteins was not observed, there is a clear diffuse and strong immunoreactivity in vCJD and iCJD samples, while in sCJD and in AD slices the reactivity was more confined to astrocytes and less pronounced (Fig. 10S)

## Discussion

In this work we provide evidence for strong up-regulation of *SERPINA3* mRNA in the CNS of patients deceased from different forms of prion diseases. A total of 128 samples were analysed from the frontal cortex of vCJD, sCJD, iCJD, gCJD, and FFI and GSS syndrome cases; as controls, AD and neurologically normal age-matched cases were used. Among the different prion diseases, there was striking up-regulation of *SERPINA3* mRNA in iCJD specimens, up to about 350 fold. Nevertheless, other forms of prion disease show significant up-regulation of the *SERPINA3* transcript, ranging from 30 to 40 folds in all groups considered. This up-regulation at the mRNA level is paralleled by enhanced SERPINA3 protein expression in astrocytes as well as in neurons. The discrepancy between mRNA and protein levels, concerning either the magnitude of up-regulation and the differences among the different groups, may depend on the half-life of the protein. While mRNA is continuously produced, the protein may be degraded quite readily, thus resulting the divergence seen between the mRNA and Western blotting results. In addition, we have to consider the different sensitivity of RT-qPCR and WB techniques, the latter being much lower in sensitivity, which could also help explain the discrepancies between mRNA and protein levels.

In addition, in an animal model of prion infection (CD1 mice intracranially inoculated with RML prions) the up-regulation of *serpina3n* and its protein product serpina3n follows the detection of prions as early as at the presymptomatic stage. Intriguingly, our data on mice inoculated with RML prions clearly show that the increase of serpina3n, particularly at the protein level but also its transcript, is concomitant with the appearance of PrP^Sc^. All inoculated animals were sacrificed at 97 dpi; all animals with PrP^Sc^ detectable in the brain by Western blot also showed evidence of serpina3n mRNA upregulation. However, the single animal that did not display detectable brain PrP^Sc^ had no evidence of serpina3n upregulation, with a mRNA level comparable to that one of the age-matched controls (Fig. 5). These data strongly suggest a direct correlation between serpina3n and prion disease progression, as previously reported also in RML-infected C57BL/6 mice^37^. In a previous work, *serpina3n* mRNA, together with GFAP mRNA, was found up-regulated in the brain of Chandler-infected SWR/j mice, since week 15 post inoculation and progressively increased to the terminal stages of the disease^38^. Similar results were observed also in C506M3 scrapie-infected and, with a minor extent, in BSE infected C57BL/6 mice^39^.

The name SERPINA3, also known as alpha 1-antichymotrypsin, comes from serine protease inhibitor class A member 3 and it belongs to the serine protease inhibitor superfamily, which comprises several members in mammals^40^. The mature protein comprises about 398 amino acids after proteolytic removal of a 25 amino acid signal sequence. The mature protein has a molecular weight of about 56–66 kDa due to heavy glycosylation^18^. The glycosylation is characteristic for circulating plasma serpins but it is dispensable for their activity^18^. The protein inhibits serine proteases and several targets have been described, including cathepsins, chymases and elastases. The mechanism by which SERPINA3 inhibits its targets is through the binding to the protease and consequent irreversible conformational change in its structure^41^. SERPINA3 has been linked to neurodegeneration and in particular to AD pathology, since protein deposits have been found in association with Aβ-peptides in both non-congophilic amorphous and classical (pre)amyloid plaques^42–45^ but also in astrocytes, in few neurons and in neurofibrillary tangles^46^. More recently, transgenic mouse models overexpressing astroglial serpina3n together with mutant amyloid protein precursor (APP), showed an increased age-related amyloid plaque deposition if compared to mice expressing APP alone. The mechanism through which this occurs it has not been yet clarified, however it is suggested that serpina3n may promote the aggregation of Aβ and its deposition into the brain parenchyma or that it could interfere with the degradation and removal of Aβ aggregates^47,48^. Despite this evidence, controversial findings have not clarified the involvement of SERPINA3 in AD pathology and its role during the course of the disease^16^. Little is known about SERPINA3 and prion diseases. One report investigated the link between a polymorphism in the signal peptide sequence of SERPINA3 and incidence of CJD, but neither allele frequencies nor genotype distribution were found altered in CJD cases if compared to controls, and also the age at onset and disease duration were unaffected by the SERPINA3 genotype^49^. More recently, *SERPINA3* has been found up-regulated in the occipital cortex of sCJD patients and the protein as well was found increased in urines^37^. How SERPINA3 is up-regulated in prion diseases remains to be established. Several intriguing hypotheses about the role of SERPINA3 in the prion replication and accumulation can be put forward. For example, SERPINA3 strong up-regulation can readily block target serine proteases and, in turn, the clearance of prions could be hampered, over time. Another captivating possibility is that increased expression of SERPINA3 could serve as a molecular chaperone which contributes to the conversion process of prion formation^50^. This hypothesis is supported by the finding of the dysregulation of *PRNP* gene, which is down-regulated in nearly all prion diseases analyzed, and again with particular relevance in iCJD samples. The exacerbation observed for *SERPINA3* mRNA in iCJD samples may be explained by the younger age of these patients (29 years on average) if compared to the other acquired from of CJD studied (vCJD, 35 years on average) who might display a stronger reactivity to the infection. However, in order to exclude any age-effect, we normalized each disease group against a specific age-matched control group. In addition, we should take into consideration that iCJD patients have been repeatedly exposed to prion-contaminated hGH over time since early childhood, through intramuscular injections which represent an invasive and highly efficient transmission route. Moreover, we also observed a great difference between the different populations within the same disease. Indeed, the up-regulation of Italian-French vCJD cases was much higher (FC = 365) than the British one (FC = 27), suggesting a potential influence of different prion strain infectivity and/or hidden susceptibility factors (such as polymorphisms) that may differentially affect the dysregulation of *SERPINA3*.

Similarly, within the iatrogenic group, the up-regulation observed in French cases (FC = 702) was much higher when compared to that one of the British samples (FC = 248), reinforcing the latter hypothesis.

Concerning the correlation between SERPINA3 levels and PrP^Sc^ pathology, we performed IHC analyses on GFAP expression, which is a well-established marker of prion disorders. Even though we did not observe a perfect correlation, we still detected a similar trend between GFAP reactivity and SERPINA3 one. This, together with the fact that we detected an up-regulation of both *gfap* and *serpina3n* mRNA also at the preclinical stage in mice, strongly point toward a correlation between SERPINA3 and PrP^Sc^ pathology.

It is likely that the up-regulation of SERPINA3 is not exactly the same in all the different brain regions, indeed it is probably more prominent in regions were astrogliosis occurs, given its predominantly astrocytic expression. However, the up-regulation is widely spread across the different brain areas, given that both RNA and protein levels were strongly up-regulated also in mice were we analyzed whole brain tissue, both for RT-qPCR and WB.

As for other tissues outside the brain, we also investigated the presence of SERPINA3 protein in the CSF where it is well expressed but with no difference between AD and sCJD samples. One reason for this lack of difference between the two diseases might depend on a possible impairment of the blood-brain barrier in AD cases, being these patients much older and the disease having a longer course, which could lead to a higher presence of SERPINA3 in the CSF of AD patients.

Previous studies have already detected a down-regulation of the *PRNP* mRNA in the frontal cortex of MM1 sCJD brains, as well as the total PrP protein content. In contrast to the present study, reduced levels of *PRNP* mRNA have also been detected in AD patients^51^. One possible explanation for this discrepancy might reside in the different stages of the disease considered, which were early (Braak stage I-II) in our study and advanced (Braak stage V-VI) in their work. Even though a direct interaction between the two proteins is still to be clarified, we can envision a mechanism in which the up-regulation of a protease-inhibitors such as SERPINA3 might lead, in turn, to an inhibition of proteases that could facilitate prion conversion, replication and accumulation.

*PRNP* mRNA down-regulation could be the consequence of an increased content of total PrP due to PrP^Sc^ accumulation, which in turn up-regulates the production of protease-inhibitors such as SERPINA3 that would further sustain the prion accumulation and propagation. Interestingly, SERPINA3 – when present in a poorly glycosylated state - is also able to retrotranslocate in the nucleus^52^ and to regulate DNA synthesis by promoting chromatin condensation^19^. Therefore we could also hypothesize that the down-regulation of *PRNP* transcript might be a direct consequence of SERPINA3 localization within the nucleus. Besides this, it is known that also PrP^Sc^ can retrotranslocate from the cytoplasm to the nucleus where it concentrates in euchromatin areas^53^. We could then suggest that upon prions accumulation, PrP^Sc^ translocates in the nucleus where it could activate *SERPINA3* transcription and subsequent SERPINA3 protein up-regulation, ultimately leading to decreased protease activity and consequent enhanced prion conversion and/or propagation. No effective treatment or cure is available for prion diseases^54^, and this lack of therapeutic approaches is further complicated by the heterogeneity and complexity of clinical presentation among different cases. With initial symptoms that span from personality changes, anxiety, depression, insomnia and impairment of autonomic functions, which worsen during the rapid course of the disease, it is difficult to determine a clinical diagnosis with certainty. Very recently, protein misfolding cyclic amplification (PMCA) technique has been successfully applied to human blood samples for the detection of minute amounts of vCJD prions. However, this approach still needs to be validated for its ability to monitor the disease at preclinical stages; indeed, only few samples from preclinical vCJD cases were available for these studies^55,56^. In addition, cerebrospinal fluid (CSF) and olfactory mucosa real-time quaking-induced conversion (RT-QuIC) has been successfully employed for detection of prions in the clinical phase of sCJD, providing virtually 100% diagnostic sensitivity and specificity^57^. In our work, we identified a potential additional biomarker highly specific for prion diseases, either sporadic, genetic of acquired. This specificity is further corroborated by *GFAP* mRNA expression pattern, which mirrors the one of *SERPINA3*. Indeed, GFAP is a well-known marker of astrogliosis, which is a hallmark of prion diseases: the fact that we find both *GFAP* and *SERPINA3* -either mRNA and protein- strongly up-regulated in the brain tissue of patients with prion disease, reinforce the hypothesis of a strong correlation between prions and SERPINA3 and support our proposal of using *SERPINA3* as a potential additional biomarker for human prion diseases. In blood *SERPINA3* mRNA is absent (Fig. 6S), therefore this tissue is not suitable for diagnostic purposes. Additional studies in other readily accessible tissues such as CSF and urine may be needed to address the diagnostic potential of *SERPINA3* mRNA detection in both preclinical and clinical stages of human prion diseases.

## Conclusions

In light of the most recent epidemiologic data reported in the Appendix III study, it is plausible that we may face a new wave of prion cases in the near future, highlighting the importance of further investigation on additional genes modulating human prion diseases.

The elevated and specific up-regulation of *SERPINA3* mRNA in human brain samples of prion diseases offers the possibility of using this transcript as a new tool to help distinguish different forms of these disorders. The ready detection of *SERPINA3* mRNA and the corresponding protein product serpina3 in CNS samples and potentially in non-CNS samples may allow us to discriminate, in different stages of the disease, various forms of prion diseases.

Understanding the molecular mechanism for SERPINA3 up-regulation in prion diseases may shed light to the cellular events that regulates prion conversion, replication and accumulation.

## Methods

### Ethics statement

All samples were collected according to the ethical and safety regulations of the Countries in which samples were collected as follows.

Human biological samples and associated data were obtained from the Austrian CJD Surveillance and KIN-Biobank Medical University of Vienna (Ethics Committe approval: 396/2011). All tissue samples were obtained according to Austrian legislations. The human tissue examined in this study was provided by the MRC Edinburgh Brain Bank and its use was covered by ethical approval from the East of Scotland Research Ethics Service REC 1 (reference number 16/ES/0084). Informed consent for the research use of autopsy tissue was obtained from the relatives of the deceased whenever necessary.

Human brain tissue was obtained from the Institute of Neuropathology Brain Bank (HUB-ICO-IDIBELL Biobank, Barcelona, Spain) following the guidelines of Spanish legislation (Real Decreto 1716/2011) and the approval of the local ethics committee.

For the French cases, written informed consent for autopsy and research use was provided by patient’s relatives, according to the French regulation (L.1232–1 to L.1232-3, Code de la Santé Publique). The brain tissues with the corresponding written informed consent were referred for postmortem diagnosis and research to the French National Neuropathological Network for CJD (funded by the French Government) and to the French National Centre of Reference for prions (funded by the French Institute for Public Health Surveillance). *PRNP* gene was analyzed pre-mortem in all cases with the informed and signed consent of the patient’s family.

This study was approved by the institutional review board of Carlo Besta Neurological Institute and performed according to the guidelines approved by the ethics committee. Written informed consent for participation in research was done in accordance with the Declaration of Helsinki (1964-2008) and the Additional Protocol on the Convention of Human Rights and Biomedicine concerning Biomedical Research (2005).

Brain tissue samples were obtained at University hospital of Verona from patients with a clinical diagnosis of prion disorders for neuropathological studies. Written informed consent was obtained for the sample included in this study.

All experiments in mice were performed in accordance with European regulations [European community Council Directive, November 24, 1986 (86/609/EEC)] and were approved by the local authority veterinary service.

### Mouse samples

Age- and sex-matched CD1 mice (Crl:CD1(ICR) strain code 022, charles river) were used for prion infection experiments. Rocky Mountain Laboratory (RML) prion infected brain homogenate was prepared at a 10% w/v in phosphate-buffered saline (PBS). 2.5 μl of 10% brain homogenate was stereotactically injected in the hippocampus of 2-months old outbred CD1 mice (n = 8). Not inoculated age- and sex-matched CD1 mice were used as controls (n = 8). Presymptomatic animals (n = 4) were sacrificed 97 days post inoculation, while terminal stage animals (n = 4) were monitored daily for clinical signs of prion disease and sacrificed at the end stage. Adult animals were sacrificed with CO_2,_ brains were extracted, immediately frozen in liquid nitrogen and stored at −80 °C. Brains of prion-infected and age-matched controls were either homogenized for WB analysis or subjected to RNA extraction.

### Patient samples

A total of 226 frontal cortex tissue samples from neurodegeneration-affected patients and healthy control subjects were collected. The samples included in our analysis (128) came from patients with sCJD (type 1 n = 15, type 2 n = 8), vCJD (n = 20), iCJD (n = 11), genetic prion diseases (FFI n = 9, gCJD n = 17, GSS n = 4), patients first diagnosed with AD as non-prion neurodegenerative surrogate controls (n = 14), and aged-matched non-neurodegenerative control cases (n = 30). All the prion disease cases have been confirmed by PrP^Sc^ detection with Western Blot or *PRNP* mutations. AD diagnosis was performed based on Braak scoring and neuropathologically confirmed. As for the controls, they died from unrelated conditions and all of them were all lacking any neurological sign. The full list of samples and patient details is reported in Table 1S.

Blood samples (n = 4) were obtained by Fondazione I.R.C.C.S Istituto Neurologico Carlo Besta.

### Tissues and RNA extraction

Total RNA was isolated from about 100 mg of frontal cortex from frozen postmortem human brain tissue or from one hemisphere of mouse whole brain tissue. The tissue was homogenized with Stainless Steel Beads 5 mm in Tissue Lyser II (Qiagen), in TRIzol reagent (Invitrogen) following manufacturer’s instructions. RNA was extracted with PureLink RNA Mini Kit (Life Technologies) and on-column DNA digestion was performed using PureLink DNase Set (Life Technologies). Blood samples were collected in PAXgene Blood RNA Tubes (Qiagen) and the related RNA was extracted using PAXgene Blood RNA Kit (Qiagen). RNA was checked for concentration and purity on a NanoDrop 2000 spectrophotometer (Thermo Scientific) whereas RNA integrity was analyzed using 2100 Bioanalyzer (Agilent Technologies). Samples included in our gene expression analysis displayed RNA Integrity Number (RIN) ≥ 4, even if some RNA samples below this threshold were selected in order to increase the sample size of very rare prion diseases patients groups.

### Reverse transcription and Real Time-quantitative PCR (RT-qPCR)

For human frontal cortex and mouse whole brain, cDNA was obtained starting from 3 µg of total RNA with 50 µM Oligo(dT)20, 10 mM dNTP mix, 5X First Strand Buffer, 0.1 M DTT, 40 U RNAse inhibitor and 200 U SuperScript^®^ III Reverse Transcriptase (Life Technologies). cDNA from blood samples was obtained using 750 ng RNA, maintaining the same reaction conditions as above. For each sample a negative control was performed by omission of the reverse transcriptase (-RT control). qPCR primers were designed using the online tool Primer-Blast provided by NCBI and, possibly, spanning an exon-exon junction or flanking intron sequence/s, to prevent the amplification of genomic DNA.

The primers sequences for human targets were as follows: for *GAPDH* 5′-CCTGCACCACCAACTGCTTA-3′ and 5′-TCTTCTGGGTGGCAGTGATG-3′; for *ACTB* 5′-AGAGCTACGAGCTGCCTGAC-3′ and 5′-AGCACTGTGTTGGCGTACAG-3′; for *RPL19* 5′-CTAGTGTCCTCCGCTGTGG-3′ and 5′-AAGGTGTTTTTCCGGCATC-3′; *B2M* for 5′-AGATGAGTATGCCTGCCGTG-3′and 5’-TCATCCAATCCAAATGCGGC-3′; for *SERPINA3* 5′ TGCCAGCGCACTCTTCATC 3′ and 5′ TGTCGTTCAGGTTATAGTCCCTC 3′; for *ALAS2* 5′-TGTCCGTCTGGTGTAGTAATGA-3′ and 5′- GCTCAAGCTCCACATGAAACT-3′.

The primers sequences for mouse targets were as follows: *Gapdh* 5′-TTCACCACCATGGAGAAGG*C*-3′ and 5′-GGCATGGACTGTGGTCATGA-3′; for *Actb* 5′-CACACCCGCCACCAGTTC-3′ and 5′-CCCATTCCCACCATCACACC-3′; for *Tubb3* 5′-CGCCTTTGGACACCTATTC-3′ and 5′-TACTCCTCACGCACCTTG-3′; for *Serpina3n* 5′-ACCCTGAGGAAGTGGAAGAA-3′ and 5′-CCTGATGCCCAGCTTTGAAA-3′.

Gene expression assays were performed using iQ^TM^ SYBR^®^ Green Supermix 2x (Bio-Rad Laboratories, Inc.), 400 nM final concentration of the corresponding forward and reverse primer (Sigma) and 10 ng/μl final concentration of cDNA samples. CFX96 Touch^TM^ Real-Time PCR Detection System (Bio-Rad Laboratories, Inc.) was used to perform the amplification and the cycling conditions included an initial denaturation of 3 min at 95 °C then 45 cycles at 95 °C for 10 sec and 60 °C for 1 min. qPCR reaction was performed in technical triplicates for each primer pair and sample. -RT sample for each primer pairs was analyzed to evaluate possible genomic RNA contamination and non-template control (NTC) was added in each plate to exclude presence of contaminating DNA in the qPCR reaction mix. Melting curve analysis of amplicons was performed for each primer pair to verify that artificial products or primer dimers were not responsible for the obtained fluorescence signals.

### RT-qPCR data analysis

Differential expression of human target genes was normalized to four reference genes (*GAPDH*, *ACTB*, *RPL19* and *B2M)* expression. For mouse samples, *Gapdh*, *Actb and Tubb3* were used as reference genes. The absolute expression value (C_T_) of a specific human erythrocyte marker, *ALAS2*, was used to account for blood contamination of brain samples. The relative expression ratio (fold change, FC) was calculated using 2^−∆∆CT^ method^58^. ∆C_T_ were calculated subtracting the C_T_ of the housekeeping gene to the C_T_ of the target one, both for “test” (disease affected patient) and “calibrator” (control). Then, ∆∆C_T_ was calculated subtracting the average ∆C_T_ of age-matched control groups to the average ∆C_T_ of each disease groups. To select age-matched control for each prion diseases and AD group, the same sample size of non-neurodegenerative controls was chosen to match both median and average age of disease affected patient groups. Matching the sex was not possible due to a higher prevalence of males in the control group. However, it has been reported that no relevant sex variability for Serpina3 exists in different disease and tissues^59,60^, therefore we are reasonably confident that this sex mismatch would not bias our results. Fold change values smaller than 1 were converted using the equation -1/fold change, for representation.

### Statistical analysis

Test-F was used to verify the homoscedasticity of variance between ∆C_Ts_ of disease-affected and the related age-matched control group (α = 0.05). Then, level of significance was calculated using unpaired student t-test (2 tails, *p* < 0.05) between ∆C_Ts_ of disease and control groups.

### Immunohistochemical analysis

Brains were fixed in 10% formalin, dehydrated and embedded in para-plast. Seven-micrometer thick serial sections were stained with hematoxylin-eosin (H&E, Bioptica) or immunostained with polyclonal antibodies to serpina3 (Sigma), monoclonal antibodies to PrP (3F4, Dako) and polyclonal antibodies to Glial Fibrillary Acidic Protein (GFAP, Dako). Before PrP immunostaining formalin-fixed samples were treated with autoclave (120 °C, 10 min), 80% formic acid (20 min) and guanidine isothiocyanate (4 M, 1 hour). Before serpina3 immunostaining all sections were treated with 80% formic acid (20 min) at room temperature. Samples were finally incubated with the Envision Plus/Horseradish Peroxidase System for rabbit and mouse immunoglobulins (DakoCytomation). Immunoreactions were visualized using 3-3′-diaminobenzidine (DAB, Dako) as chromogen.

### Production and purification of recombinant human SERPINA3

Plasmid pET-11° containing the sequence coding for HuSerA3 (GenScript) was transformed into Escherichia coli RosettaGami cells for the expression. Cells were grown in LB medium at 37 °C in presence of ampicillin (100 µg/mL) until OD = 600 nm, followed by induction with 0,5 mM IPTG overnight. Cells were collected by centrifugation and resuspended into Cell Lysis Buffer (25 mM Tris, 0,8% Triton X-100). Cells were disrupted using PANDA Homogenizer, and inclusion bodies containing the desired protein were subsequently isolated by centrifugation. Inclusion bodies were subjected to a series of washing steps, and then solubilized overnight at 37 °C in 8 M GdnHCl, 25 mM Tris. The purification process takes advantage of the His tag added at the C-terminal of the protein. The inclusion bodies were filtered and then applied onto a HisTrap column (GE Healthcare). The unspecific proteins were washed away with 5 column volumes of Binding Buffer, and then HuSerA3 was eluted with Elution Buffer, containing 500 mM imidazole. HuSerA3 fractions with higher purity were pooled together and refolded with serial dialysis steps to reduce the amount of denaturing agent. The protein was then dialysed against PBS and stored at −20 °C.

### Immunoblot analysis

Frontal cortex of patients with sporadic (sCJD), genetic (gCJD, E200K), iatrogenic CJD (iCJD), Alzheimer’s disease (AD) and healthy controls were homogenized in lysis buffer (NaCl 100 mM, EDTA 10 mM, NP40 0.5%, Deoxycholic acid 0.5%, TRIS 10 mM pH 7.4) at 10% weight/volume. Samples were centrifuged (Eppendorf Centrifuge) for 1 minute at 4 °C (800 × g) to remove cellular debris. Two-hundred µg of proteins were loaded onto 12% Bis/Tris Acrylamide gels (NuPAGE, Invitrogen) and separated by SDS-Page, transferred to PVDF membrane and incubated with polyclonal anti-serpin3 rabbit antibody (1:500 Sigma-Aldrich), 3F4 antibody (1:10,000 Dako) and β-actin (1:10,000 Sigma-Aldrich). The reactions were visualized by chemiluminescence using Amersham ECL Prime (GE Healthcare Life Sciences). For PrP analysis, samples were digested with 50 µg/mL of proteinase-K (Life Technologies) at 37 °C for 1 hour. Densitometric analysis was carried out using UVIBand software.

For mouse samples the same procedure as above was followed. Detection of PrP^Sc^ was performed using W226 monoclonal antibody (1:1000) and a goat anti-mouse HRP secondary antibody. Actin beta was detected using monoclonal anti-β-actin−peroxidase antibody (1:10,000 Sigma), SerpinA3n detection was performed using Mouse Serpin A3N Antibody (1:500 R&D Systems) and a rabbit anti-goat HRP secondary antibody.

### Data availability

All data generated and/or analyzed during the current study are available from the corresponding author on reasonable request.

### Compliance with Ethical Standards

#### Ethical approval

All procedures performed in studies involving human participants were in accordance with the ethical standards of the institutional and/or national research committee and with the 1964 Helsinki declaration and its later amendments or comparable ethical standards. All human samples were anonymized. (for further details see Materials and Methods section).

All procedures performed in studies involving animals were in accordance with the ethical standards of the institution or practice at which the studies were conducted. Current animal husbandry and housing practices comply with the Council of Europe Convention ETS123 (European Convention for the Protection of Vertebrate Animals used for Experimental and Other Scientific Purposes; Strasbourg, 18.03.1986); Italian Legislative Decree 26/2014, Gazzetta Ufficiale della Repubblica Italiana, 26 July 2014; and with the 86/609/EEC (Council Directive of 24 November 1986 on the approximation of laws, regulations and administrative provisions of the Member States regarding the protection of animals used for experimental and other scientific purposes). Animal facility is licensed and inspected by the Italian Ministry of Health. Mice were housed in groups of 2–5 animals in individually ventilated cages, daily fed and water provided ad libitum. Lighting was on an automatic 12 hours basis. All surgery was performed under tribromoethanol anesthesia, and all efforts were made to minimize suffering and regular veterinary care was daily performed for assessment of animal health. The study, including its Ethics aspects, was approved by the Italian Ministry of Health (Permit Number: NP-02-14).

#### Informed consent

Informed written consent was obtained for all individual participants included in the study. (for details see Materials and Methods section).

## Electronic supplementary material

Supplemetary figures and table

## References

[1] Prusiner SB (2013). Biology and genetics of prions causing neurodegeneration. Annu Rev Genet.

[2] Collee JG, Bradley R, Liberski PP (2006). Variant CJD (vCJD) and bovine spongiform encephalopathy (BSE): 10 and 20 years on: part 2. Folia Neuropathol.

[3] Mok T (2017). Variant Creutzfeldt-Jakob Disease in a Patient with Heterozygosity at PRNP Codon 129. N Engl J Med.

[4] Gill ON (2013). Prevalent abnormal prion protein in human appendixes after bovine spongiform encephalopathy epizootic: large scale survey. BMJ.

[5] Advisory Committee on Dangerous Pathogens TSE Risk Assessment Subgroup (August 2016). “Appendix-III” position statement. Available from: ACDP TSE subgroup minutes, agendas and papers, https://app.box.com/s/hhhhg857fjpu2bnxhv6e (2016).

[6] Hilton DA (2004). Prevalence of lymphoreticular prion protein accumulation in UK tissue samples. J Pathol.

[7] Kim MO, Geschwind MD (2015). Clinical update of Jakob-Creutzfeldt disease. Current opinion in neurology.

[8] Llewelyn CA (2004). Possible transmission of variant Creutzfeldt-Jakob disease by blood transfusion. Lancet.

[9] Peden AH, Head MW, Ritchie DL, Bell JE, Ironside JW (2004). Preclinical vCJD after blood transfusion in a PRNP codon 129 heterozygous patient. Lancet.

[10] Wroe SJ (2006). Clinical presentation and pre-mortem diagnosis of variant Creutzfeldt-Jakob disease associated with blood transfusion: a case report. Lancet.

[11] Peden A (2010). Variant CJD infection in the spleen of a neurologically asymptomatic UK adult patient with haemophilia. Haemophilia.

[12] Colby, D. W. & Prusiner, S. B. *Prions*. *Cold Spring Harb Perspect Biol* **3**, a006833, doi:3/1/a006833 [pii] 10.1101/cshperspect.a006833 (2011). 10.1101/cshperspect.a006833 PMC3003464 21421910

[13] DeArmond, S. J. & Bouzamondo, E. Fundamentals of prion biology and diseases. *Toxicolog*y **181–182**, 9–16, doi:S0300483X02002494 [pii] (2002). 10.1016/s0300-483x(02)00249-4 12505278

[14] Gasperini L, Legname G (2014). Prion protein and aging. Front Cell Dev Biol.

[15] Barbisin M (2014). Gene expression profiling of brains from bovine spongiform encephalopathy (BSE)-infected cynomolgus macaques. BMC Genomics.

[16] Baker C, Belbin O, Kalsheker N, Morgan K (2007). SERPINA3 (aka alpha-1-antichymotrypsin). Front Biosci.

[17] Miranda E, Lomas DA (2006). Neuroserpin: a serpin to think about. Cell Mol Life Sci.

[18] Hwang SR, Steineckert B, Kohn A, Palkovits M, Hook VY (1999). Molecular studies define the primary structure of alpha1-antichymotrypsin (ACT) protease inhibitor in Alzheimer’s disease brains. Comparison of act in hippocampus and liver. J Biol Chem.

[19] Naidoo N, Cooperman BS, Wang ZM, Liu XZ, Rubin H (1995). Identification of lysines within alpha 1-antichymotrypsin important for DNA binding. An unusual combination of DNA-binding elements. J Biol Chem.

[20] Elliott PR, Pei XY, Dafforn TR, Lomas DA (2000). Topography of a 2.0 A structure of alpha1-antitrypsin reveals targets for rational drug design to prevent conformational disease. Protein Sci.

[21] Mahadeva R (2001). Association of alpha(1)-antichymotrypsin deficiency with milder lung disease in patients with cystic fibrosis. Thorax.

[22] Koomen JM (2005). Plasma protein profiling for diagnosis of pancreatic cancer reveals the presence of host response proteins. Clin Cancer Res.

[23] Leinonen J, Lovgren T, Vornanen T, Stenman UH (1993). Double-label time-resolved immunofluorometric assay of prostate-specific antigen and of its complex with alpha 1-antichymotrypsin. Clin Chem.

[24] Higashiyama M (1995). Alpha-1-antichymotrypsin expression in lung adenocarcinoma and its possible association with tumor progression. Cancer.

[25] Yamamura J (2004). mRNA expression level of estrogen-inducible gene, alpha 1-antichymotrypsin, is a predictor of early tumor recurrence in patients with invasive breast cancers. Cancer Sci.

[26] Abraham CR, Selkoe DJ, Potter H (1988). Immunochemical identification of the serine protease inhibitor alpha 1-antichymotrypsin in the brain amyloid deposits of Alzheimer’s disease. Cell.

[27] Fraser PE, Nguyen JT, McLachlan DR, Abraham CR, Kirschner DA (1993). Alpha 1-antichymotrypsin binding to Alzheimer A beta peptides is sequence specific and induces fibril disaggregation *in vitro*. J Neurochem.

[28] Janciauskiene S, Rubin H, Lukacs CM, Wright HT (1998). Alzheimer’s peptide Abeta1-42 binds to two beta-sheets of alpha1-antichymotrypsin and transforms it from inhibitor to substrate. J Biol Chem.

[29] Sun YX, Wright HT, Janciauskiene S (2002). Alpha1-antichymotrypsin/Alzheimer’s peptide Abeta(1-42) complex perturbs lipid metabolism and activates transcription factors PPARgamma and NFkappaB in human neuroblastoma (Kelly) cells. J Neurosci Res.

[30] Wang Q (2012). Stability of endogenous reference genes in postmortem human brains for normalization of quantitative real-time PCR data: comprehensive evaluation using geNorm, NormFinder, and BestKeeper. Int J Legal Med.

[31] Stan AD (2006). Human postmortem tissue: what quality markers matter?. Brain research.

[32] Schoor O (2003). Moderate degradation does not preclude microarray analysis of small amounts of RNA. Biotechniques.

[33] Koppelkamm A, Vennemann B, Lutz-Bonengel S, Fracasso T, Vennemann M (2011). RNA integrity in post-mortem samples: influencing parameters and implications on RT-qPCR assays. Int J Legal Med.

[34] Fleige S, Pfaffl MW (2006). RNA integrity and the effect on the real-time qRT-PCR performance. Mol Aspects Med.

[35] Weis S (2007). Quality control for microarray analysis of human brain samples: The impact of postmortem factors, RNA characteristics, and histopathology. J Neurosci Methods.

[36] Penna I (2011). Selection of candidate housekeeping genes for normalization in human postmortem brain samples. Int J Mol Sci.

[37] Miele G (2008). Urinary alpha1-antichymotrypsin: a biomarker of prion infection. PLoS One.

[38] Campbell IL, Eddleston M, Kemper P, Oldstone MB, Hobbs MV (1994). Activation of cerebral cytokine gene expression and its correlation with onset of reactive astrocyte and acute-phase response gene expression in scrapie. J Virol.

[39] Dandoy-Dron F (2000). Enhanced levels of scrapie responsive gene mRNA in BSE-infected mouse brain. Brain Res Mol Brain Res.

[40] Silverman GA (2001). The serpins are an expanding superfamily of structurally similar but functionally diverse proteins. Evolution, mechanism of inhibition, novel functions, and a revised nomenclature. J Biol Chem.

[41] Huntington JA, Read RJ, Carrell RW (2000). Structure of a serpin-protease complex shows inhibition by deformation. Nature.

[42] Abraham CR, Shirahama T, Potter H (1990). Alpha 1-antichymotrypsin is associated solely with amyloid deposits containing the beta-protein. Amyloid and cell localization of alpha 1-antichymotrypsin. Neurobiol Aging.

[43] Abraham CR, Selkoe DJ, Potter H, Price DL, Cork LC (1989). Alpha 1-antichymotrypsin is present together with the beta-protein in monkey brain amyloid deposits. Neuroscience.

[44] Rozemuller JM (1991). Distribution pattern and functional state of alpha 1-antichymotrypsin in plaques and vascular amyloid in Alzheimer’s disease. A immunohistochemical study with monoclonal antibodies against native and inactivated alpha 1-antichymotrypsin. Acta Neuropathol.

[45] Verga L (1989). Alzheimer patients and Down patients: cerebral preamyloid deposits differ ultrastructurally and histochemically from the amyloid of senile plaques. Neurosci Lett.

[46] Gollin PA, Kalaria RN, Eikelenboom P, Rozemuller A, Perry G (1992). Alpha 1-antitrypsin and alpha 1-antichymotrypsin are in the lesions of Alzheimer’s disease. Neuroreport.

[47] Mucke L (2000). Astroglial expression of human alpha(1)-antichymotrypsin enhances alzheimer-like pathology in amyloid protein precursor transgenic mice. Am J Pathol.

[48] Nilsson LN (2001). Alpha-1-antichymotrypsin promotes beta-sheet amyloid plaque deposition in a transgenic mouse model of Alzheimer’s disease. J Neurosci.

[49] Salvatore, M. *et al*. Alpha1 antichymotrypsin signal peptide polymorphism in sporadic Creutzfeldt-Jakob disease. *Neurosci Lett* **227**, 140-142, doi:S030439409700308X [pii] (1997). 10.1016/s0304-3940(97)00308-x 9180223

[50] Zsila F (2010). Inhibition of heat- and chemical-induced aggregation of various proteins reveals chaperone-like activity of the acute-phase component and serine protease inhibitor human alpha(1)-antitrypsin. Biochem Biophys Res Commun.

[51] Llorens F (2013). PrP mRNA and protein expression in brain and PrP(c) in CSF in Creutzfeldt-Jakob disease MM1 and VV2. Prion.

[52] Santamaria M (2013). Nuclear alpha1-antichymotrypsin promotes chromatin condensation and inhibits proliferation of human hepatocellular carcinoma cells. Gastroenterology.

[53] Mange A, Crozet C, Lehmann S, Beranger F (2004). Scrapie-like prion protein is translocated to the nuclei of infected cells independently of proteasome inhibition and interacts with chromatin. J Cell Sci.

[54] Bolognesi ML, Legname G (2015). Approaches for discovering anti-prion compounds: lessons learned and challenges ahead. Expert opinion on drug discovery.

[55] Concha-Marambio L (2016). Detection of prions in blood from patients with variant Creutzfeldt-Jakob disease. Sci Transl Med.

[56] Bougard D (2016). Detection of prions in the plasma of presymptomatic and symptomatic patients with variant Creutzfeldt-Jakob disease. Sci Transl Med.

[57] Bongianni M (2017). Diagnosis of Human Prion Disease Using Real-Time Quaking-Induced Conversion Testing of Olfactory Mucosa and Cerebrospinal Fluid Samples. JAMA Neurol.

[58] Livak KJ, Schmittgen TD (2001). Analysis of relative gene expression data using real-time quantitative PCR and the 2(-Delta Delta C(T)) Method. Methods.

[59] Dimberg J (2011). Expression of the serine protease inhibitor serpinA3 in human colorectal adenocarcinomas. Oncol Lett.

[60] Luo D (2017). Serpin peptidase inhibitor, clade A member 3 (SERPINA3), is overexpressed in glioma and associated with poor prognosis in glioma patients. Onco Targets Ther.

