# Supplementary material for: Differential overexpression of SERPINA3 in human prion diseases
Source: Sci Rep. 2017 Nov 15;7:15637. doi: 10.1038/s41598-017-15778-8 (PMC5688139; doi:10.1038/s41598-017-15778-8)
Supplement: Supplementary file 1 — Supplemetary figures and table [file 41598_2017_15778_MOESM1_ESM.pdf]

SUPPLEMENTARY MATERIAL for

Differential overexpression of *SERPINA3* in human prion diseases

Vanni S, Moda F, Zattoni M, Bistaffa E, De Cecco E, Rossi M, Giaccone G, Tagliavini F, Haïk S, Deslys JP, Zanusso G, Ironside JW, Ferrer I, Kovacs GG and Legname G

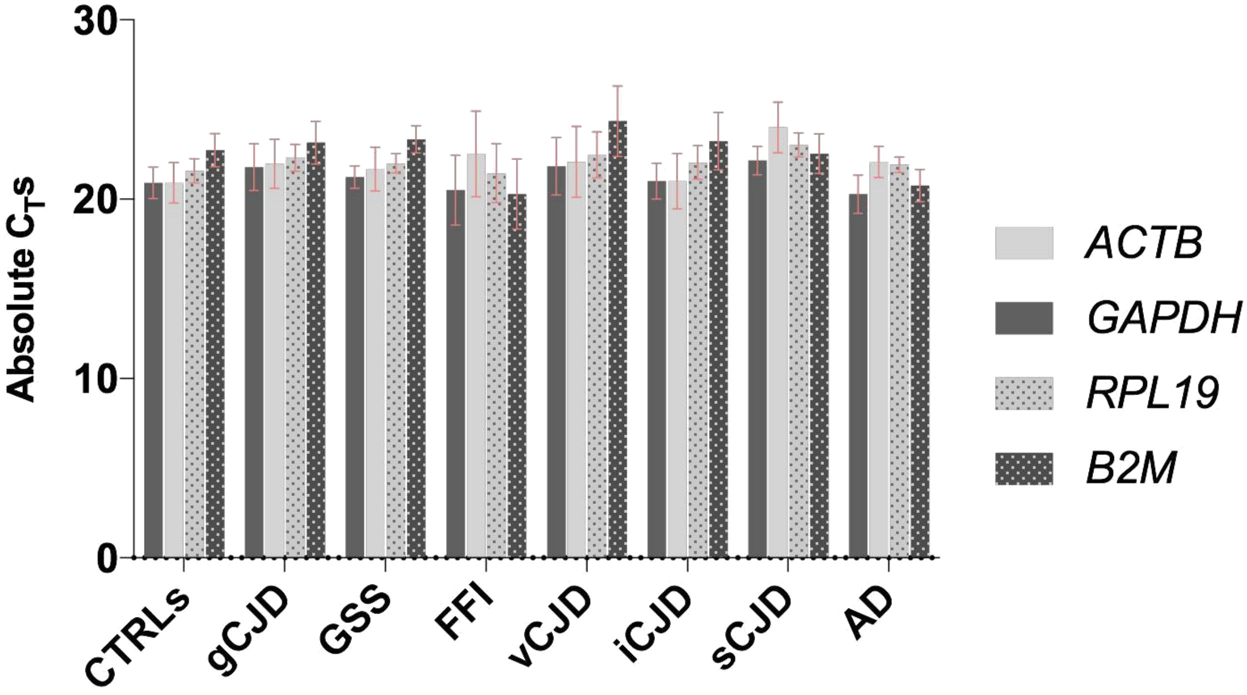

Figure 1S Comparison between *GAPDH*, *ACTB, RPL19* and *B2M* transcripts stability across control and neurodegenerative diseases affected patients. For each group, average values of absolute C_{Ts} among the samples are shown. Each sample was analyzed in triplicates.

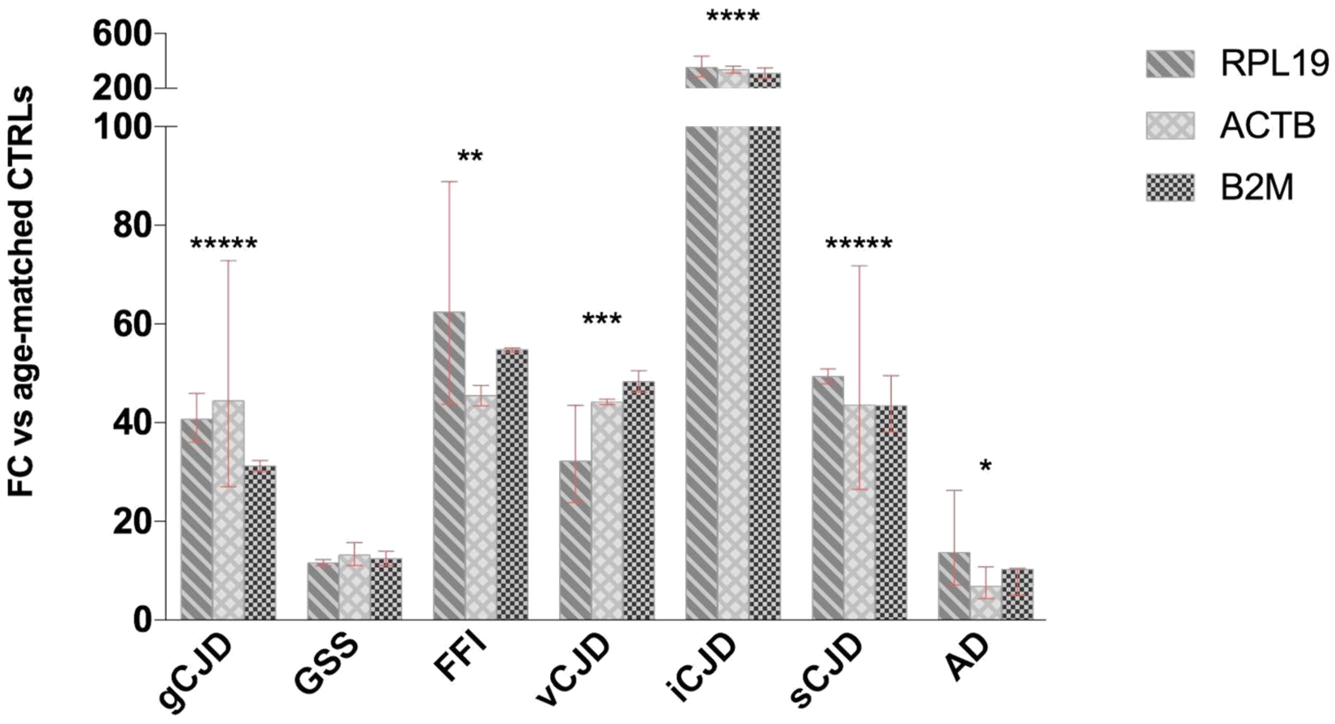

Figure 2S RT-qPCR of *SERPINA3* VS *RPL19-ACTB-B2M*. FC are showed as averages normalized against each reference gene, with related CI. Dashed columns show normalization against *RPL19*, light dotted gray normalization against *ACTB* and dark dotted gray normalization against *B2M*. gCJD (n=17) *****p<0.000000005, FFI (n=9) **p<0.0005, vCJD (n=20) ***p<0.0000001, iCJD (n=11) ****p<0.00000001, sCJD (n=23) *****p<0.000000005, AD (n=14) *p<0.005).

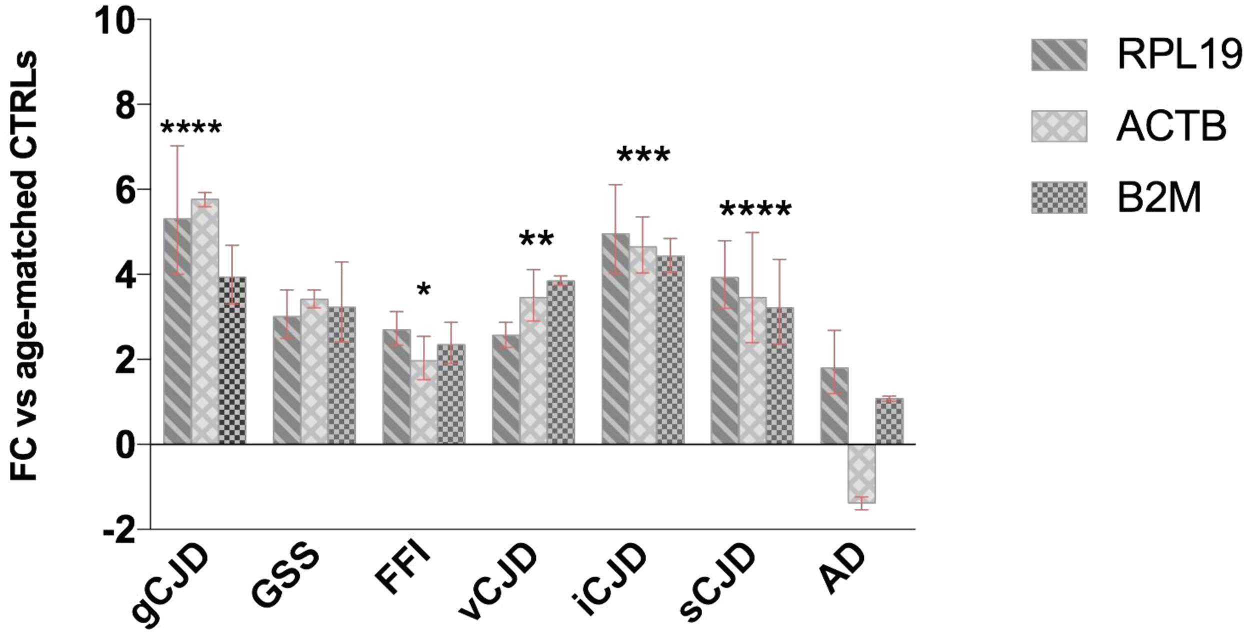

Figure 3S RT-qPCR of *GFAP* VS *RPL19-ACTB-B2M*. FC are showed as averages normalized against each reference gene, with related CI. Dashed columns show normalization against *RPL19*, light dotted gray normalization against *ACTB* and dark dotted gray normalization against *B2M*. gCJD (n=17) ****p<0.00005, FFI (n=9) *p<0.05, vCJD (n=20) **p<0.0005, iCJD (n=11) ***p<0.0001, sCJD (n=23) ****p<0.00005.

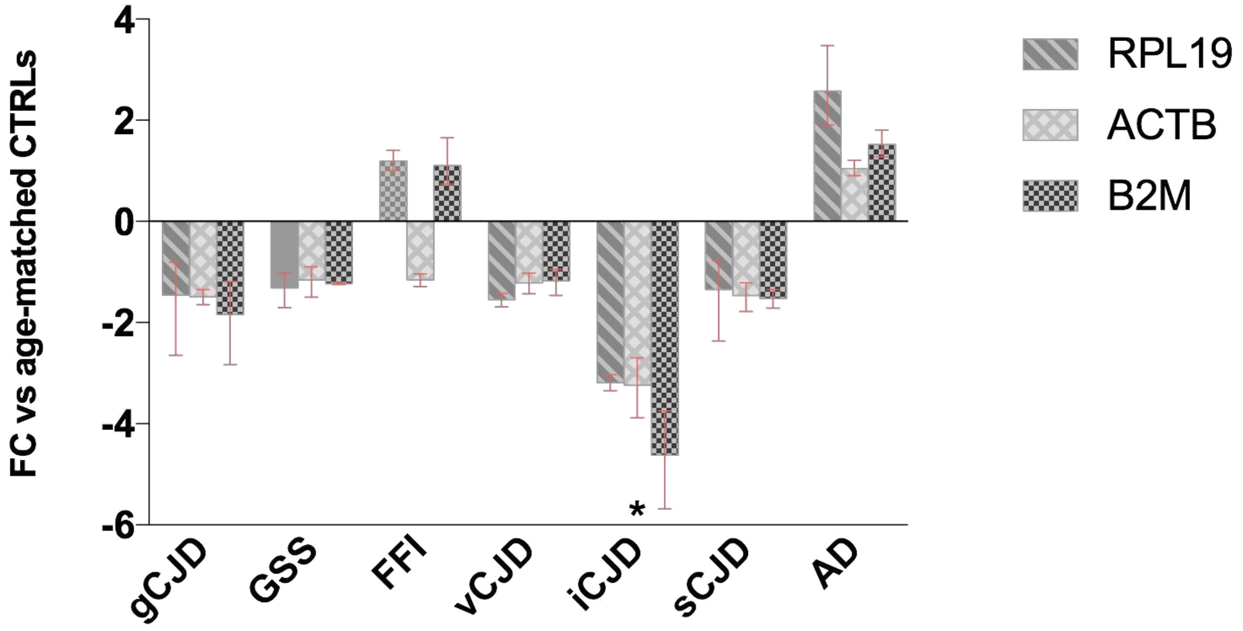

Figure 4S RT-qPCR of *PRNP* VS *RPL19-ACTB-B2M*. FC are showed as averages normalized against each reference gene, with related CI. Dashed columns show normalization against *RPL19*, light dotted gray normalization against *ACTB* and dark dotted gray normalization against *B2M*. iCJD (n=11) *p<0.0001

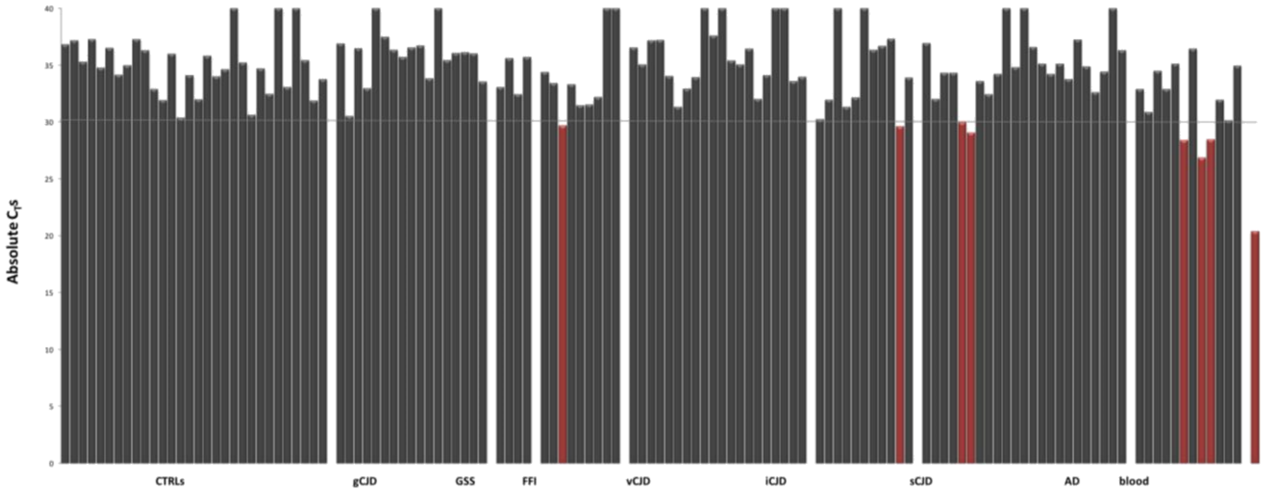

Figure 5S Erythrocyte marker expression across controls and patients. Absolute C_T values for *ALAS2* in control and neurodegenerative affected brain samples. Blood from two healthy individuals is used as positive control.

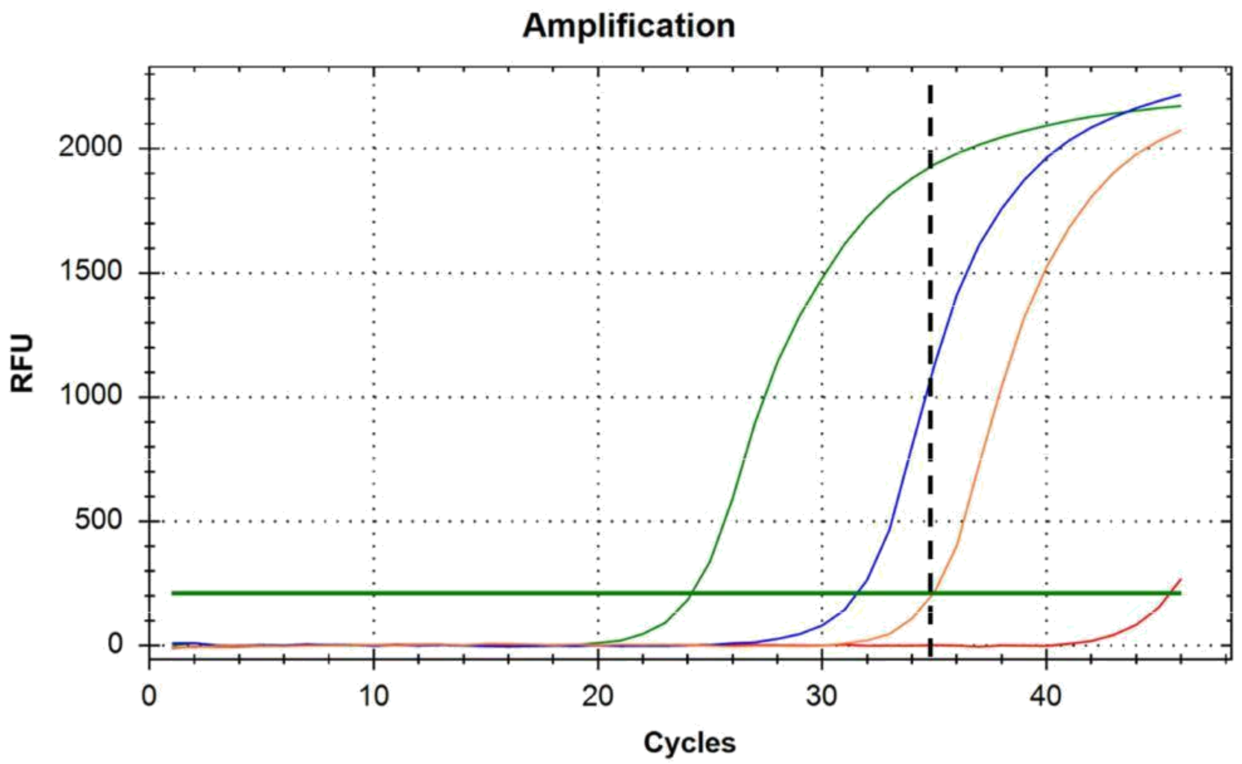

Figure 6S RT-qPCR amplification plot for target genes in human whole blood. Absolute quantification of *ALAS2* (green), *PRNP* (blue), *GFAP* (orange) and *SERPINA3* (red) presence in 1 ng of cDNA of human whole blood from two sCJD infected patients and two healthy controls. Dashed line indicates the absence of transcripts threshold.

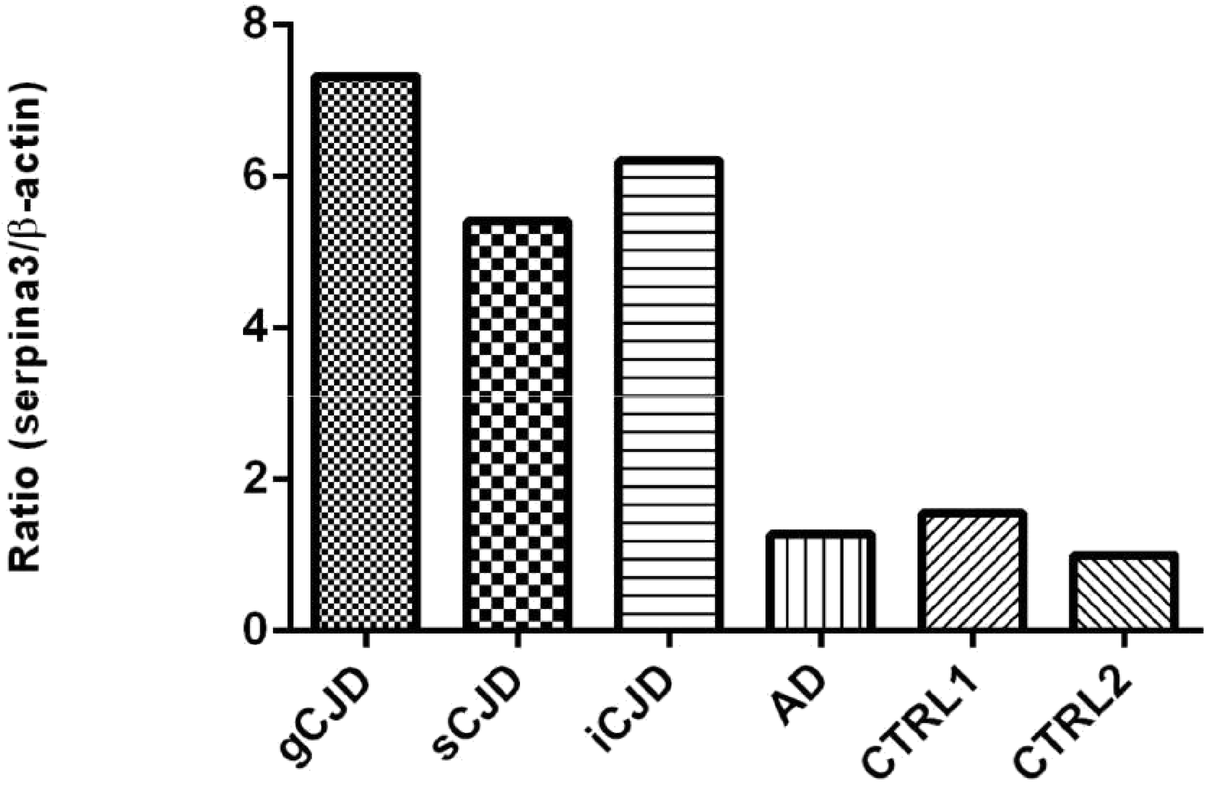

Figure 7S Densitometric quantification of serpina3. Densitometric analysis of Western blot of serpina3 shown in Fig. 4 were performed and revealed that the level of expression of the protein was about four to six-folds upregulated in CJD samples compared to AD samples and to healthy controls.

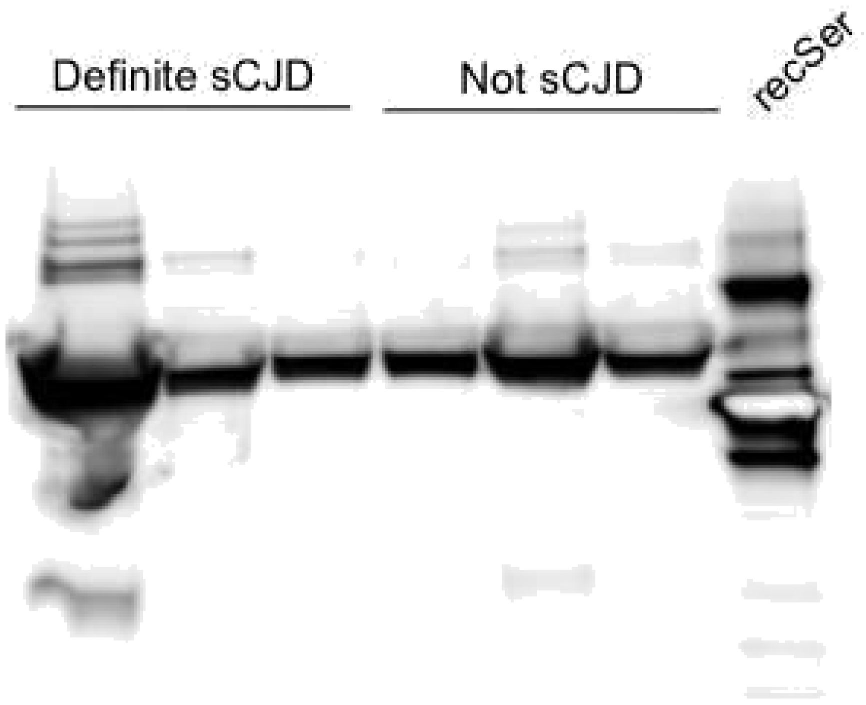

Figure 8S Western blotting analysis of CSF samples from sCJD and non-sCJD patients. The expression levels of SERPINA3 in CSF samples of representative cases from each group were assessed by means of Western blotting. Rec lane refers to recombinant SERPINA3 used as control.

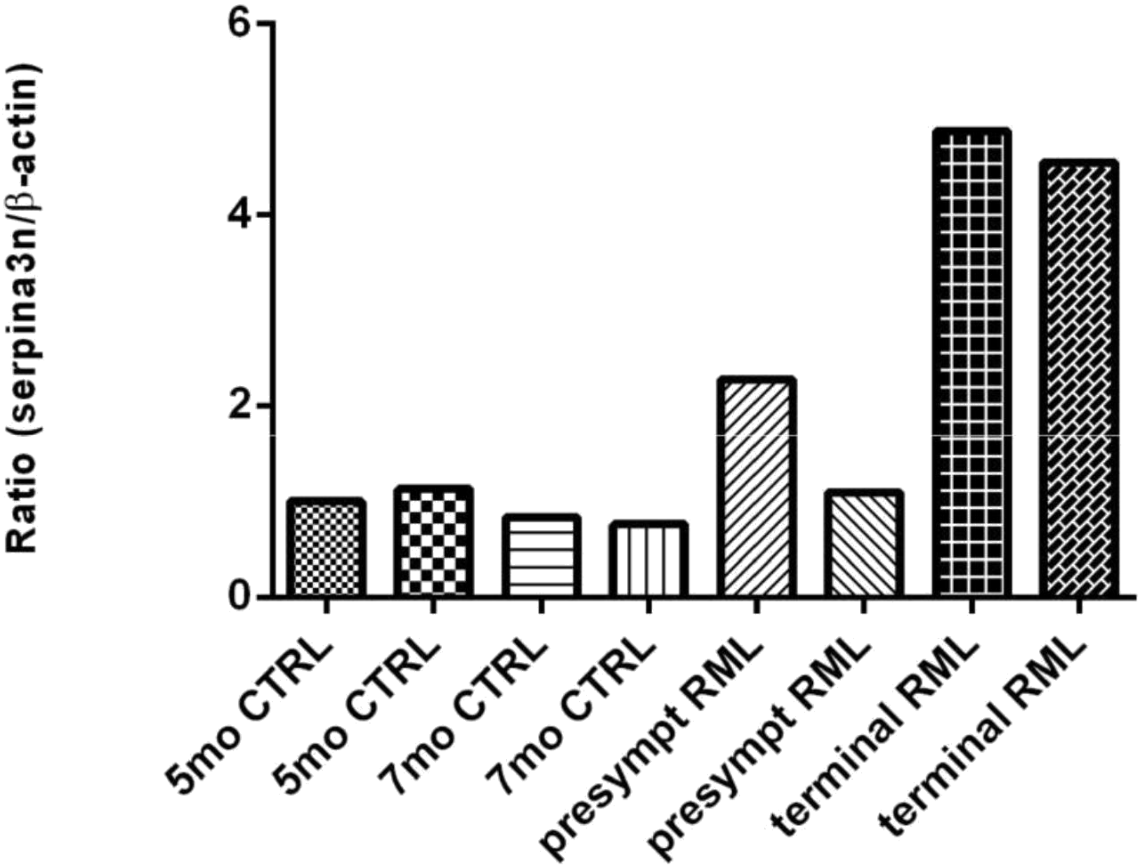

Figure 9S Densitometric quantification of serpina3n. Densitometric analysis of Western blot of serpina3n levels shown in Fig. 6 were performed and revealed that the level of expression of the protein was about two-fold times upregulated in presymptomatic RML infected mice and six-fold higher in terminal RML infected mice, compared to their related age-matched controls.

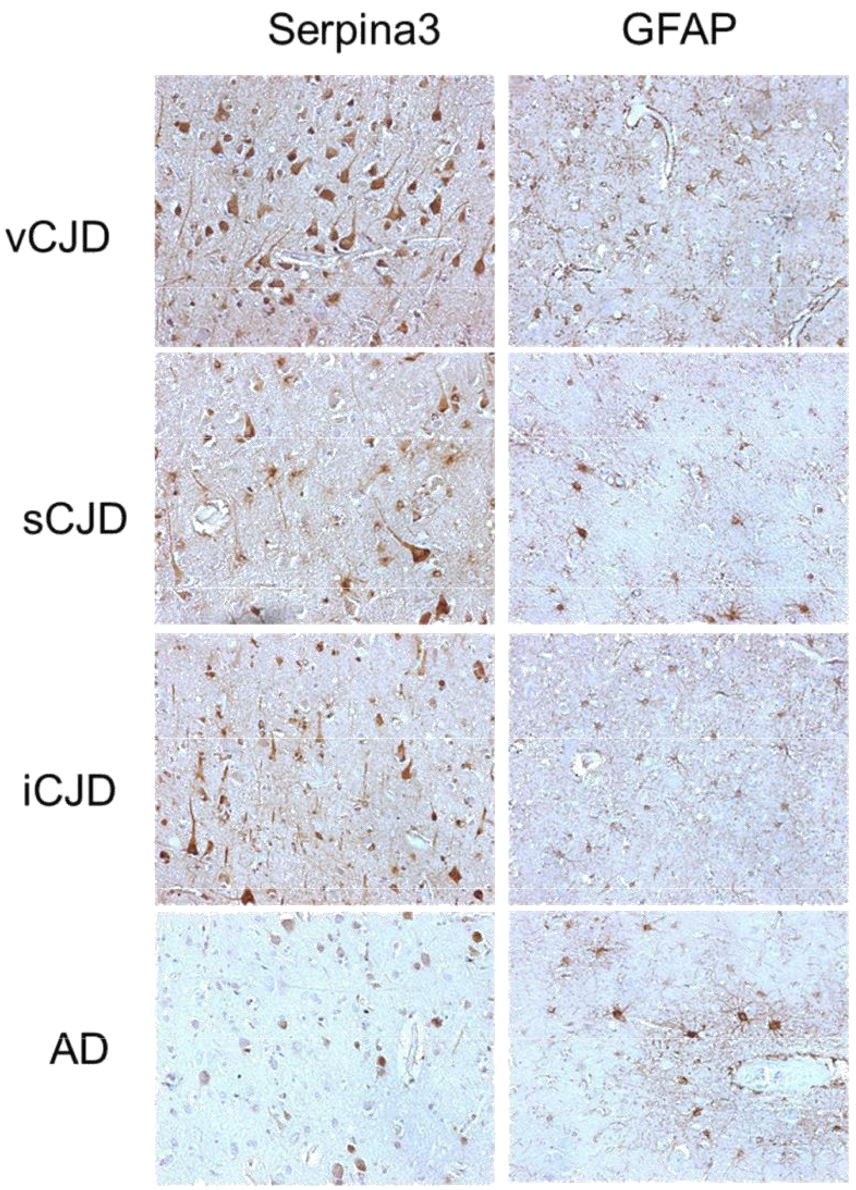

Figure 10S Neuropathological analysis of serpina3 and gfap. Immunohistochemical analysis revealed strong serpina3 immunoreaction in the frontal cortex of CJD patients (variant, sporadic and iatrogenic forms) while was only faint in AD patients. Similarly, gfap immunoreactivity was strong and diffused in vCJD and iCJD samples while more faint in sCJD and AD slices.

Case number	Gender	Age	Codon 129	*PRNP* mutation	Braak stage
**CTRL**					
2338	M	16	nd		
26976	M	19	nd		
2467	M	25	nd		
2367	M	25	nd		
2518	M	27	nd		
7270	M	29	nd		
2569	M	31	nd		
24342	M	33	nd		
21396	M	38	nd		
3765	M	39	nd		
20592	M	43	nd		
18393	F	46	nd		
26797	M	49	nd		
4176	M	51	nd		
19687	F	51	nd		
15221	M	53	nd		
3783	M	56	nd		
24781	M	57	nd		
24780	F	57	nd		
18391	M	58	nd		
7628	M	60	nd		
22612	M	61	nd		
18407	M	62	nd		
20121	M	63	nd		
13410	M	68	nd		
14395	F	71	nd		
9508	M	76	nd		
1656	F	62	nd		
1682	F	59	nd		
17/14	M	76	nd		

Case number	Gender	Age	Codon 129	*PRNP* mutation	Braak stage
**gCJD**					
249	M	51	MM	E200K	
250	M	66	MV	E200K	
252	F	55	MM	E200K	
1066	M	56	VV	V210I	
254	F	56	nd	E200K	
257	F	42	MM	E200K	
258	M	63	MM	4XINS	
259	M	53	MM	E211Q	
7260	F	53	MM	E200K	
24522	M	78	VV	E200K	
24525	F	56	nd	E200K	
170-15	M	73	MM	E200K	
186-15	F	61	MV	E200K	
116-14	F	60	MV	E200K	
46-16	F	73	MV	E200K	
008/47	F	58	M/V	5-opri	
008/13	M	39	M/V	4-opri	
**FFI**					
123-11	F	49	MV	D178N-M	
74-05	F	63	MM	D178N-M	
67-02	F	39	MM	D178N-M	
169-96	M	26	MM	D178N-M	
184-97	M	37	MM	D178N-M	
7/490	F	42	M/M	D178N-M	
008/80	F	64	M/V	D178N-M	
8/179	F	48	M/M	D178N-M	
1358	F	64	nd	D178N-M	
**GSS**					
28713	F	48	MM	P102L	
282	F	45	nd	P102L	
2060	M	60	MV	P84S	
24338	F	49	MM	Q212P	

Case number	Gender	Age	Codon 129	*PRNP* mutation	Braak stage
**vCJD**					
1229	F	25	MM		
1559	F	42	MM		
289	M	36	MM		
1067	M	30	MM		
640	F	58	MM		
1432	F	33	MM		
229	F	37	MM		
007/96	M	30	MM		
045/96	M	31	MM		
110/96	F	35	MM		
148/98	M	20	MM		
154/98	M	36	MM		
100/99	F	17	MM		
129/99	M	33	MM		
51/03	M	18	MM		
56/03	M	62	MM		
110/03	M	30	MM		
029/05	F	32	MM		
112/05	F	34	MM		
046/10	M	62	MM		
**iCJD**					
2137	F	31	MM		
2057	M	25	MM		
388	F	27	MM		
1937	M	36	MV		
286	F	24	MV		
84/94	M	25	VV		
245/97	F	27	MM		
246/97	M	36	VV		
29/99	M	27	VV		
111/19	M	37	MV		
73/03	M	30	nd		

Case number	Gender	Age	Codon 129	*PRNP* mutation	Braak stage
**sCJD**					
1679	M	60	M/M		
1669	M	72	M/M		
9/230	M	77	M/M		
7/396	M	25	M/M		
1508	F	53	M/M		
1407	F	79	nd		
1728	F	61	nd		
1368	M	64	M/M		
9/001	M	83	M/M		
1722	M	59	nd		
1723	F	86	M/M		
1675	M	64	M/V	208A	
1524	F	78	M/V		
1620	F	63	nd		
9/214	M	71	M/M		
1548	M	65	M/M		
1504	M	77	V/V		
1645	M	68	nd		
1665	F	76	nd		
008/38	F	51	V/V		
9/297	F	82	M/V		
008/69	M	65	V/V		
1370	F	72	nd		
**AD**					
A10/46	M	74	nd		AD, BS II
A11/13	M	70	nd		AD, BS I
A10/64	M	86	nd		AD, BS II
A10/45	M	67	nd		AD, BS I
A10/6	M	57	nd		AD, BS II
A11/75	M	61	nd		AD, BS I
A10/98	F	73	nd		AD, BS I
A11/51	M	58	nd		AD, BS I
A10/27	M	68	nd		AD, BS I
A10/77	M	65	nd		AD, BS II
A11/55	M	60	nd		AD, BS II
A10/34	M	64	nd		AD, BS I
1677	M	90	nd		AD, BS III
1721	M	74	nd		AD, BS I

Table 1S Main details of control, gCJD, GSS, FFI, vCJD, iCJD, sCJD and AD cases included in the present study.
F female, M male, *MM* methionine/methionine, *MV* methionine/valine, *VV* valine/valine, *BS* Braak stage
